# Syntenin Controls Extracellular Vesicle‐Induced Tumour Migration by Regulating the Expression of Adhesion Proteins on Small Extracellular Vesicles

**DOI:** 10.1002/jev2.70133

**Published:** 2025-08-20

**Authors:** Barnabas Irmer, Allegra Angenendt, Luc Camoin, Stéphane Audebert, Christiane Geyer, Mirjam Gerwing, Hanna Spiessbach, Mira Hebel, Émilie Baudelet, Darius Wlochowitz, Uwe Hansen, Annalen Bleckmann, Pascale Zimmermann, Kerstin Menck

**Affiliations:** ^1^ Department of Medicine A, Hematology, Oncology, and Pneumology University of Muenster Muenster Germany; ^2^ West German Cancer Center University Hospital Muenster Muenster Germany; ^3^ Marseille Proteomics Platform, Centre de Recherche en Cancérologie de Marseille (CRCM) Institut Paoli‐Calmettes, Aix‐Marseille Université, Inserm, CNRS Marseille France; ^4^ Centre de Recherche en Cancérologie de Marseille (CRCM) Institut Paoli‐Calmettes, Aix‐Marseille Université, Inserm, CNRS Marseille France; ^5^ Clinic for Radiology University of Muenster Muenster Germany; ^6^ Department of Medical Bioinformatics University Medical Center Goettingen Goettingen Germany; ^7^ Institute for Musculoskeletal Medicine University of Muenster Muenster Germany; ^8^ Department of Human Genetics K. U. Leuven Leuven Belgium; ^9^ Clinic for Hematology/Medical Oncology University Medical Center Goettingen Goettingen Germany

**Keywords:** adhesion, breast cancer, cell migration, extracellular vesicles, integrins, Syntenin

## Abstract

Despite extensive proof for the tumour‐supporting function of cancer‐derived small extracellular vesicles (sEVs), attributions of pathological effects to specific sEV subpopulations are poorly described. In this study, we aimed to characterise a distinct sEV species under the control of Syntenin, a key regulator of endosomal sEV biogenesis, regarding its proteomic cargo and pro‐tumourigenic functions. Using mass spectrometry (MS), we detected 178 down‐ and 236 up‐regulated proteins on sEVs from breast cancer cells upon Syntenin knockout (KO). Pathway enrichment analysis suggested that Syntenin depletion was particularly associated with adhesion‐related processes. Accordingly, sEVs from Syntenin‐deficient 4T1 and MCF‐7 breast cancer cells showed a reduced expression of several focal adhesion and cell–cell junction proteins. Syntenin silencing reduced the Fibronectin‐binding capacity of sEVs from both cell lines, which was mediated by sEV‐associated Integrin alpha‐V/beta‐3 (α_V_β_3_). Compared to sEVs from wildtype cells, Syntenin KO sEVs showed decreased tropism towards the Fibronectin‐rich liver microenvironment in vivo, provided less adhesive support for 4T1 cells and thereby failed to induce cancer cell migration, which appeared to be independent of EV uptake. In summary, this study revealed that Syntenin has a large‐scale effect on the proteomic cargo of sEVs and regulates their adhesive, organotropic and pro‐migratory properties in breast cancer.

**Abbreviations**
CTF: C‐terminal fragment
ECM: extracellular matrix
EVs: extracellular vesicles
FCS: fetal calf serum
GO: Gene Ontology
KD: knockdown
KO: knockout
lEV: large extracellular vesicle
MS: mass spectrometry
NTA: nanoparticle tracking analysis
RT: room temperature
sEV: small extracellular vesicle
TEM: transmission electron microscopy
WT: wildtype

## 1 Introduction

Syntenin/*SDCBP* (syndecan binding protein), also known as MDA‐9 (melanoma differentiation‐associated gene‐9), is an intracellular scaffolding protein that harbours two PDZ domains. While its basal expression in adult tissues is rather low, an upregulation was detected in various tumours, including glioblastoma, melanoma, colorectal, lung, prostate, and breast cancer (Pintor‐Romero et al. 2023). A high expression of Syntenin correlated with increased tumour growth and metastasis (Yang et al. 2013; Liu et al. 2018). On the cellular level, Syntenin has been associated with various cancer‐related processes, such as cell adhesion, migration, growth, proliferation, or immune evasion (Zimmermann et al. 2001; Kashyap et al. 2015; Liu et al. 2018). Moreover, Syntenin was identified as a key regulator for the biogenesis of a specific subclass of extracellular vesicles (EVs), the endosomal‐derived exosomes (Baietti et al. 2012).

EVs are small lipid nanoparticles that can transport a plethora of cellular components, thus mediating intercellular communication over short and long distances. In cancer, tumour EVs can either act as paracrine signalling factors educating tumour‐adjacent stromal cells (e.g. fibroblasts, immune cells, endothelial cells) or target cancer cells in an autocrine fashion, thereby inducing tumour invasion and migration (Lucotti et al. 2022). Additionally, EVs can enter the circulation, which on the one hand facilitates their usage as fertile sources for cancer‐specific biomarkers via liquid biopsies (Irmer et al. 2023a), and on the other hand contributes to the formation of pre‐metastatic niches (Peinado et al. 2012; Hoshino et al. 2015).

Based on their size, EVs are roughly categorised into large EVs (lEVs, diameter > 150 nm) and small EVs (sEVs, diameter < 150 nm). Syntenin is almost exclusively expressed on sEVs (Kowal et al. 2016), most likely due to its contribution to exosome biogenesis: Upon binding to the C‐terminus of plasma membrane‐associated proteoglycans of the Syndecan (SDC) family (Grootjans et al. 1997), Syntenin was shown to recruit the ESCRT (endosomal‐sorting complex required for transport)‐associated protein ALIX. Together, they are implicated in the budding of late endosomal membranes which results in the formation of intraluminal vesicles (ILVs) inside multivesicular endosomes (Baietti et al. 2012). Once these cell organelles fuse with the plasma membrane, the ILVs are released into the extracellular space as so‐called ‘exosomes’, a specific subpopulation of sEVs with a diameter <200 nm (Welsh et al. 2024). Previous data have demonstrated that oncogenic SRC signalling, often observed in human tumours, can induce the generation of exosomes by phosphorylating Syntenin and SDCs (Imjeti et al. 2017). These exosomes, in turn, can support the migration of endothelial cells (Imjeti et al. 2017) and thus link Syntenin to an aggressive sEV phenotype.

Still, sEVs as mediators for Syntenin‐associated tumour progression remain poorly investigated. Despite reported protein interactions between Syntenin and sEV‐associated proteins like SDC4, ALIX, CD63, and SRC (Baietti et al. 2012; Imjeti et al. 2017), the influence of Syntenin on the large‐scale proteomic cargo of sEVs has not been addressed yet. In this study, we therefore aimed to reveal the proteomic profile of Syntenin‐regulated sEVs and to address its relevance for the pro‐tumoural phenotype of breast cancer‐derived sEVs. We demonstrate that Syntenin loss‐of‐function is associated with a loss of adhesion proteins on sEVs, which are important for anchoring the sEVs to the extracellular matrix (ECM). As a consequence, Syntenin‐negative sEVs show a profound reduction in their potential to stimulate breast cancer migration.

## 2 Methods

### 2.1 Cell Culture and Transfections

Human MCF‐7 (DSMZ) and murine 4T1 (ATCC) breast cancer cells were kept under normal cell culture conditions (37°C, 95% humidity, 5% CO_2_) in DMEM/F12 or RPMI‐1640, respectively, supplemented with 10% heat‐inactivated (56°C, 30 min) fetal calf serum (FCS). 4T1 cells with stable knockout (KO) of Syntenin were generated by transiently transfecting cells with the PX461‐GFP and ‐mCh plasmid encoding for the Cas9n (D10A nickase mutant) with sgRNAs targeting *Sdcbp* exon 3 (KO_1, sequences: 5’‐AGCATTTGTTCTGGTGGATG‐3’ and 5’‐CTCTCCCTCCAGATGGAAGT‐3; KO_2, sequences: 5’‐GGCTCAAACTGCTTATTCTG‐3’ and 5’‐CAGCCAAGCATTTGTTCTGG‐3’). The pSpCas9n(BB)‐2A‐GFP (PX461‐GFP) vector was a gift from Feng Zhang (Addgene plasmid #48140; http://n2t.net/addgene:48140; RRID: Addgene_48140) (Ran et al. 2013), and the pSpCas9n(BB)‐2A‐mCherry (PX461‐mCh) was cloned by substituting GFP with monomeric Cherry (mCh). Double‐positive cells were sorted by flow cytometry and grown as single‐cell clones. MCF‐7 cells with transient knockdown (KD) of Syntenin were generated by transfecting cells at 80% density with siRNA (final concentration: 10 nM) using the MISSION siRNA reagent (Sigma). Cells were used 72 h post‐transfection for downstream analyses. Human siRNA for Syntenin, including a non‐targeting control (siCTL), was purchased from Dharmacon. Target sequences (5’‐3’) were siCTL: AUGUAUUGGCCUGUAUUAG, KD_1: GCAAGACCUUCCAGUAUAA, KD_2: GAAGGACUCUCAAAUUGCA. The dual approach of using two cell lines and two distinct gene silencing methods enabled us to confirm the observed effects across different cell lines and species, while also minimising the risk of off‐target effects or compensatory mechanisms that may arise from stable genetic modifications.

### 2.2 Antibodies

Primary antibodies are listed in Table S1. The antibody for murine Syntenin has been previously described in (Baietti et al. 2012). Secondary antibodies were anti‐mouse‐HRP (#7074, 1:20,000) and anti‐rabbit‐HRP (#7076, 1:20,000, both from CST) for immunoblots and anti‐mouse AF594 (#405326, 1:2000) and anti‐rabbit AF488 (#406416, 1:2000, both from Biolegend) for immunofluorescence. The antibody against human Integrin alpha‐V/beta‐3 (#MAB3050) and the respective IgG1 control antibody (#MAB002, both from R&D Systems) were used as blocking antibodies for functional assays.

### 2.3 Preparation of EVs

For the isolation of EVs from cell culture supernatants, cells were washed twice with PBS and cultured for 24 h in medium supplemented with 10% EV‐depleted FCS (ultracentrifuged for 18 h at 150,000 × *g* with subsequent filtration through a 0.2 µm filter [Shelke et al. 2014]). Conditioned medium was collected and after removal of cells (500 × *g*, 5 min) and debris (1500 × *g*, 15 min), lEVs (17,000 × *g*, 30 min) and sEVs (143,000 × *g*, 90 min) were pelleted in a XPN‐80 ultracentrifuge (Sw32Ti rotor, Beckman Coulter). EVs were washed once in PBS and stored in 30–100 µL PBS at −20°C. EV protein concentrations were measured by Dc Protein Assay Kit (Bio‐Rad) according to the manufacturer's instructions. For the additional purification of EVs by density gradient ultracentrifugation, 150–400 µg of isolated EVs in 1 mL PBS were layered on top of a 5%–40% iodixanol (OptiPrep, Sigma) gradient buffered in Tris/EDTA (pH 7.4) and were centrifuged for 18 h at 100,000 × *g* in a XPN‐80 ultracentrifuge (Sw32.1Ti rotor, Beckman Coulter) as described previously (Schöne et al. 2024). Seventeen fractions were collected, washed in PBS for 1 h at 100,000 × *g* in a Max‐XP ultracentrifuge (TLA‐55 rotor, Beckman Coulter) and pellets were used for immunoblot analysis. All relevant EV isolation and characterisation data were uploaded to the EV‐TRACK knowledgebase (EV‐TRACK ID: EV240031) (Van Deun et al. 2017).

### 2.4 EV Labelling

For animal experiments, EVs were stained with 2 µM DiR (1,1‐dioctadecyl‐3,3,3,3‐tetramethylindotricarbocyanine iodide, #D12731, Thermo Scientific) in PBS for 15 min at RT and centrifuged for 1 h at 143,000 × *g* as described previously (Gerwing et al. 2020; Irmer et al. 2023b). The pelleted sEVs were resuspended in 100 µL PBS and used for injection. For adhesion and uptake assays, EVs were labelled with DiR, or PKH26 (Sigma) according to the manufacturer's instructions. Each experiment comprised a dye‐only control without EVs.

### 2.5 Transmission Electron Microscopy

EVs were fixed overnight at 4°C using 2% (v/v) formaldehyde and 2.5% (v/v) glutaraldehyde in 100 mM cacodylate buffer (pH 7.4). After washing with PBS, EVs were post‐fixed with 0.5% (v/v) osmium tetroxide and 1% (w/v) potassium hexacyanofer­rate (III) in 0.1 M cacodylate buffer for 2 h. After washing with distilled water and subsequent dehydration through exposure to increasing ethanol concentrations (30%–100%), propylene oxide was used for two 15 min incubation steps. All samples were embedded in Epon, cut into ultrathin sections using an ultramicrotome and collected on copper grids. Finally, samples were negatively stained with 2% uranyl acetate for 10 min. The acquisition of electron micrographs at 60 kV was performed with a Philips EM 410 with a Veleta camera plus additional Radius software system (Emiss).

### 2.6 Nanoparticle Tracking Analysis

For the measurement of EV particle concentration and size, samples were diluted in PBS (final particle concentration: 50–400 counts per frame) and injected into the Zetaview PMX120‐S (ParticleMetrix). Signals were visualised with a 640 nm laser and a connected CMOS camera at eleven positions within the flow chamber (1 s/position). Size and concentration parameters were calculated by the ZetaView software (v8.02.31).

### 2.7 Immunoblot

To prepare cell lysates for immunoblot analysis, cells were washed once in PBS and lysed with a cell scraper in RIPA buffer (50 mM Tris, 150 mM NaCl, 0.1% SDS, 0.5% sodium deoxycholate, 1% Triton X‐100, pH 7.2) supplemented with protease (Sigma) and phosphatase (Roche) inhibitors. Equal protein amounts (up to 50 µg) of cell lysate or EVs were heated in Laemmli buffer (Bio‐Rad) and loaded onto SDS‐PAGE gels (8%–12%). Proteins were blotted onto nitrocellulose, followed by membrane blocking in TBST +3% BSA for 1 h at RT. Signals were detected after incubation with the primary antibodies overnight (ovn) at 4°C and respective secondary antibodies for 1 h at RT using West Pico (Thermo Scientific) or Clarity Max (Bio‐Rad) ECL substrate at the ChemoStar Touch Imager (Intas). Protein expression was quantified by densitometry using ImageJ software (v1.52p) and, in the case of protein signals from cell lysates, normalised to the expression of a housekeeping protein.

### 2.8 Processing of EV Samples for Mass Spectrometry

Purified EVs were loaded on NuPAGE 4%–12% Bis–Tris acrylamide gels according to the manufacturer's instructions (Life Technologies). Running of protein was stopped as soon as proteins stacked in a single band. Protein containing bands were stained with Imperial Blue (Pierce), cut from the gel and digested with high sequencing grade trypsin (Promega) before mass spectrometry (MS) analysis. Briefly, gel pieces were washed and destained using few steps of 100 mM NH_4_HCO_3_. Destained gel pieces were shrunk with 100 mM ammonium bicarbonate in 50% acetonitrile and dried at RT. Protein spots were then rehydrated using 10 mM DTT in 25 mM ammonium bicarbonate pH 8.0 for 45 min at 56°C. This solution was replaced by 55 mM iodoacetamide in 25 mM ammonium bicarbonate pH 8.0 and the gel pieces were incubated for 30 min at RT in the dark. They were washed twice in 25 mM ammonium bicarbonate and finally shrunk by incubation for 5 min with 25 mM ammonium bicarbonate in 50% acetonitrile. The resulting alkylated gel pieces were dried at RT. The dried gel pieces were reswollen by incubation in 25 mM ammonium bicarbonate pH 8.0 supplemented with 12.5 ng/µL trypsin (Promega) for 1 h at 4°C and then incubated overnight at 37°C. Peptides were harvested by collecting the initial digestion solution and carrying out two extractions; first in 5% formic acid and then in 5% formic acid in 60% acetonitrile. Pooled extracts were dried down in a centrifugal vacuum system. Samples (injected in duplicate) were reconstituted in 0.1% TFA 4% acetonitrile and analysed by LC‐MS/MS using a Q Exactive Mass Spectrometer online with a nanoLC Ultimate 3000 chromatography system (ThermoFisher Scientific). For each biological sample, 5 µL corresponding to 5% of initial sample were injected in duplicate into the system. After pre‐concentration and washing of the sample on a Acclaim PepMap 100 column (C18, 2 cm × 100 µm i.d. 100 Å pore size, 5 µm particle size), peptides were separated on a PepMap RSLC column (C18, 50 cm × 75 µm i.d., 100 Å, 2 µm particle size) at a flow rate of 300 nL/min with a three steps linear gradient (4%–20% acetonitrile/H_2_O; 0.1% formic acid for 220 min; 20%–38% acetonitrile/H_2_O; 0.1% formic acid for 75 min and 38%–55% acetonitrile/ H_2_O; 0.1% formic acid for 25 min). The separation of the peptides was monitored by a UV detector (absorption at 214 nm). For peptide ionisation in the nanospray source, spray voltage was set at 1.5 kV and the capillary temperature at 275°C. All samples were measured in a data dependent acquisition mode. Each run was preceded by a blank MS run in order to monitor system background. The peptide masses were measured in a survey full scan (scan range 300–1700 *m/z*, with 70 K FWHM resolution at *m/z* = 400, target AGC value of 1×10^6^ and maximum injection time of 120 ms). Following the high‐resolution full scan in the Orbitrap, the 12 most intense data‐dependent precursor ions were successively fragmented in HCD cell and measured in Orbitrap (normalised collision energy of 25%, activation time of 10 ms, target AGC value of 5 × 10^5^, maximum injection time 250 ms, isolation window 2 *m/z*, 17.5 K FWHM resolution). Parent masses obtained in orbitrap analyser were automatically calibrated on the 445.120025 ion used as lock mass. Dynamic exclusion was implemented with a repeat count of 1 and exclusion duration of 40 s.

### 2.9 Processing of MS Data

Relative intensity‐based label‐free quantification was processed using the MaxLFQ algorithm from the freely available MaxQuant computational proteomics platform (v1.5.3.8) (Tyanova et al. 2016). The acquired raw LC Orbitrap MS data were first processed using the integrated Andromeda search engine. Spectra were searched against a UniProt Mouse database (v2015.08) implemented of a bovine database generated from serum contamination (483 entries). The following parameters were used for searches: (i) trypsin allowing cleavage before proline; (ii) two missed cleavages were allowed; (iii) monoisotopic precursor tolerance of 20 ppm in the first search used for recalibration, followed by 4.5 ppm for the main search and 0.5 Da for fragment ions from MS/MS; (iv) cysteine carbamidomethylation as a fixed modification and methionine oxidation and N‐terminal acetylation as variable modifications; (v) a maximum of five modifications per peptide allowed; and (vi) minimum peptide length of 7 amino acids and a maximum mass of 4600 Da. The ‘match between runs’ option was enabled to transfer identifications across different LC‐MS/MS replicates based on their masses and retention time using default parameters. The quantification was performed using a minimum ratio count of 1 (unique + razor) and the second peptide option to allow identification of two co‐fragmented co‐eluting peptides with similar masses. The false discovery rate (FDR) at the peptide and protein levels was set to 1% and determined by searching a reverse database. The statistical analysis was done with Perseus (v1.5.1.6) (Tyanova and Cox 2018). The LFQ‐normalised intensities were uploaded from the proteinGroups.txt file. First, proteins marked as contaminants, reverse hits, and ‘only identified by site’ were discarded. Protein LFQ intensities were base 2 logarithmised to obtain a normal distribution. Missing values were replaced using data imputation by randomly selecting from a normal distribution centred on the lower edge of the intensity values that simulates signals of low‐abundant proteins using default parameters. Two‐sample *t*‐tests were done using permutation‐based FDR controlled at 0.05 and employing 250 permutations. The *p* value was adjusted using a scaling factor s0, and all differentially expressed proteins with *p* < 0.05 were considered significant. Among the differentially expressed proteins, we detected three viral proteins (ENV1, ENV2, POL1) that were downregulated in Syntenin KO sEVs. The reactivation of endogenous retroviruses has been observed in many mouse tumour cell lines and can lead to the co‐isolation of viral‐like particles in murine tumour sEV preparations (Cocozza et al. 2023).

### 2.10 Animal Experiments

The in vivo analysis of sEV biodistribution was carried out in female BALB/c mice from Charles River (age 8–12 weeks). Animals were kept in groups of max five animals at 23°C–26°C, 45%–65% relative humidity and a 12 h light‐dark cycle with ad libitum access to food and water as well as to play and embedding material. To minimise the risk of artefact detection in the final readout, the back and the bottom of the mice were dehaired with a razor and depilatory cream 24 h prior to the injection. A 100 µg of DiR‐labelled sEVs were intravenously injected into the tail vein of narcotised mice, and 24 h post‐injection, the mice were sacrificed to measure the DiR fluorescence in the distinct organs by fluorescence reflectance imaging (FRI) scans as described previously (Gerwing et al. 2020).

### 2.11 EV‐to‐ECM Adhesion

Glass coverslips were coated with PBS, Fibronectin (#sc‐29011), Collagen IV (#sc‐29010), or Laminin (#sc‐29012, all from Santa Cruz) for 1 h at RT in a 24‐well plate. Subsequently, the coated coverslips were incubated for 4 h at 37°C with PKH26‐labelled sEVs (2 µg/well) or respective dye‐only controls. For blocking experiments, PKH26‐labelled sEVs were pre‐treated with 20 µg/ml of the Integrin αVβ3 blocking or IgG1 control antibody for 30 min at 37°C. The coverslips were washed once with PBS and fixed in 4% PFA. All coverslips were mounted in fluoroshield mounting medium (Abcam) and imaged by confocal laser scanning microscopy using an LSM800 (Zeiss). Area fractions of thresholded PKH26 signals were quantified for each image with the ImageJ software (v1.52p).

### 2.12 Cell‐to‐EV Adhesion

PBS alone or PBS‐diluted sEVs (1 µg/well) were seeded into a tissue culture 96‐well plate (#83.3924.005, Sarstedt). After 4 h of incubation, unbound PBS or sEVs were removed, and 4 × 10^4^ cells were seeded onto the coated wells for 10 min at 37°C. The wells were washed once with PBS to remove all unbound cells. The adherent cells at the well bottom were fixed using 4% PFA and stained with 0.1% crystal violet solution diluted in 20% methanol (20 min, RT), followed by three PBS washing steps and oven drying. The dye was dissolved in 10% acetic acid (5 min, RT), and the absorption at 570 nm was measured using an Infinite 200 Pro plate reader (Tecan).

### 2.13 Migration and Immunofluorescence Assay

The wells of a 24‐well plate (#83.3922, Sarstedt) were coated with sEVs (10 µg/well) in PBS or with PBS alone for 4 h at 37°C. Unbound PBS or sEVs were removed, and 2 × 10^4^ cancer cells were seeded on top. Cell migration was observed by live cell imaging 2 h post‐seeding using a BZ‐X810 microscope (Keyence) connected to a stage‐top incubator (Ibidi). Thirty pictures per hour were captured at two positions per well for 8 h at 37°C and 5% CO_2_. Cell migration of ten representative single‐cells per position was manually tracked with ImageJ (v1.52p). To investigate cancer cell migration upon EV uptake inhibition, 4T1 cells were pre‐incubated with dansylcadaverine (180 µM) for 2 h prior to seeding on sEV‐coated plates, and treatment was continued during live cell imaging. For the analysis of Integrin downstream signalling in migrating cancer cells, 1 × 10^5^ 4T1 cells were seeded on sEV‐coated glass coverslips in 24‐well plates for 2 h. Cells were washed with PBS, fixed in 4% PFA and blocked/permeabilised with PBS + 3% BSA + 0.3% Triton X‐100 (30 min, RT). Phosphorylated FAK (pFAK Y397) and SRC (pSRC Y416) were stained with corresponding primary and AF488‐labelled secondary antibodies for 2 h or 1 h at RT, respectively. Glass coverslips were mounted in fluoroshield mounting medium with DAPI (Abcam) and imaged by confocal laser scanning microscopy using a LSM800 (Zeiss).

### 2.14 EV Uptake Studies

Glass coverslips were coated with PKH26‐labelled sEVs (2 µg/well) in a 24‐well plate for 4 h at 37°C. Plates were washed once with PBS and 1 × 10^5^ 4T1 WT cells were seeded on top. After incubating for 2 h at 37°C, the cells were washed with PBS, fixed in 4% PFA and blocked/permeabilised with PBS + 0.3% BSA + 0.05% saponin (30 min, RT). Endosomes were stained for LAMP2 for 2 h at RT followed by incubation with an AF488‐labelled anti‐rabbit antibody for 1 h at RT. Samples were mounted in fluoroshield mounting medium with DAPI (Abcam) and investigated by confocal laser scanning microscopy on a LSM800 (Zeiss). For the quantification of intracellular EV uptake, Z‐projections were created illustrating the maximal PKH26 intensity within intracellular substacks. Subsequently, the percentual area of PKH26 signals within cellular regions was measured for each image. Image analysis was performed with the ImageJ software (v1.52p). To block EV uptake, the following inhibitors were used: dansylcadaverine (Da, 180 µM, #HY‐D1027, MedChemExpress), dynasore (Dy, 10 µM, #sc‐202592, Santa Cruz), filipin III (Fi, 35 µM, #F4767, Sigma) and simvastatin (Si, 50 µM, #CS‐2269, ChemScene LLC). Viability upon inhibitor treatment was determined by MTT assay according to standard protocols. To measure the efficiency of EV uptake inhibition, 2.5 × 10^5^ 4T1 cells were seeded in a 6‐well plate and pre‐treated with the respective inhibitors for 2 h prior to the addition of DiR‐labelled 4T1 WT sEVs (10 µg/well). After 24 h, cells were washed twice with PBS and the percentage of DiR‐positive cells recorded on a FACSymphony A1 flow cytometer (BD). Data were analysed with FlowJo (v10.6.1, BD).

### 2.15 Statistics and Visualisation

Each experiment was performed in at least three independent biological replicates. Differences between multiple groups were tested for significance using a one‐way ANOVA with Fisher's LSD test, unless indicated otherwise. For comparisons between groups with more than ten data points, outliers were identified and excluded according to the ROUT method (Q = 5%). Differences were stated significant for *p* values <0.05 and marked as *p < 0.05, **p<0.01, ***p<0.001, ****p<0.0001 and ns = not significant. Statistical analyses and visualisation of all bar charts and violin plots were done with GraphPad Prism (v8.4.2), microscopic images were arranged in OMERO (v.5.14.1), and schematic illustrations were created with BioRender.com. Heatmaps were created and visualised with *Heatmapper* (Babicki et al. 2016) using Spearman rank clustering for distance measuring. Volcano plots were created with *VolcaNoseR* (Goedhart and Luijsterburg 2020).

## 3 Results

### 3.1 Characterisation of Breast Cancer‐Derived EVs

To isolate breast cancer‐derived EVs, we chose the human MCF‐7 and murine 4T1 as model cell lines. EVs were isolated from conditioned medium using differential ultracentrifugation, which results in a high yield of particles with medium purity (Welsh et al. 2024) and remains the gold standard for the isolation of different EV subpopulations. sEVs isolated from both cell lines were smaller in size compared to the respective lEVs, as shown by transmission electron microscopy (TEM) and nanoparticle tracking analysis (NTA) (Figure S1A,B). Secretion rates of sEVs per cell highly exceeded lEVs (Figure S1C). Both EV fractions were strongly enriched for respective marker proteins (ARF6 and RGAP1 for lEVs; TSG101, Syntenin and CD81 for sEVs [Kowal et al. 2016; Lischnig et al. 2022]) (Figure S1D). The absent expression of nuclear HDAC1 and Golgi‐associated GM130 excluded major contaminations with non‐EV proteins. Taken together, these data confirmed the successful isolation of the distinct EV subpopulations from the two breast cancer cell lines.

### 3.2 Syntenin Loss‐of‐Function Has a Major Impact on the Proteome of sEVs

**FIGURE 1:**
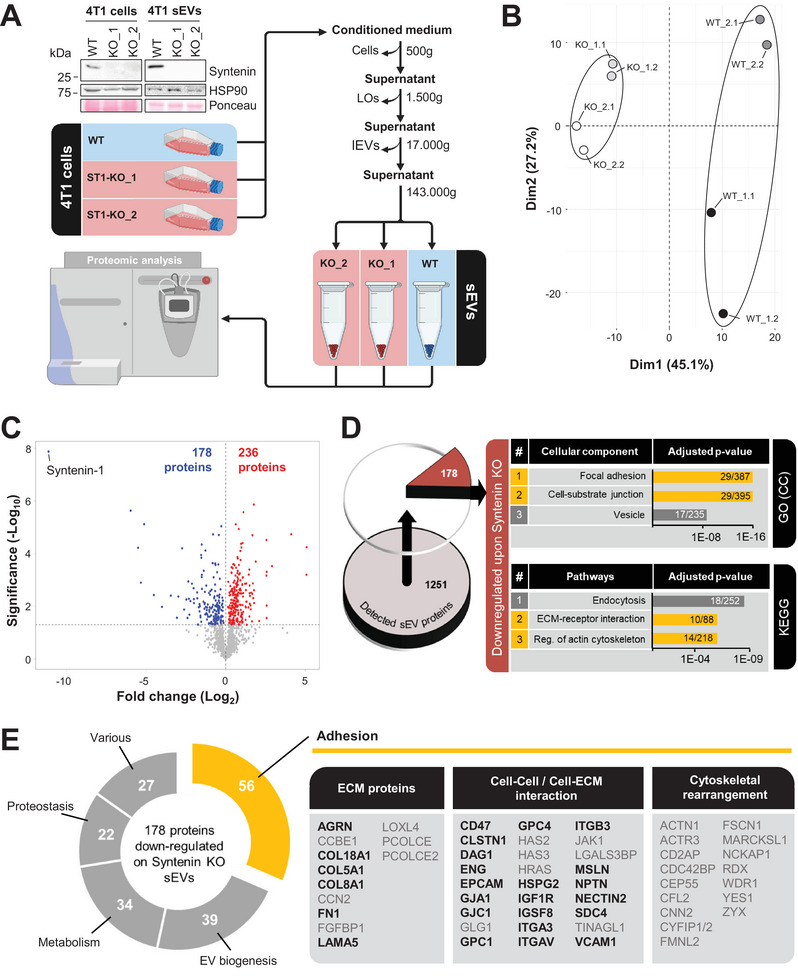
Syntenin controls the proteomic cargo of sEVs. (A) Workflow for the analysis of the sEV proteome from 4T1 wild‐type (WT) and Syntenin knockout (KO_1/2: 2 clones) cells by tandem label‐free mass spectrometry (MS). A representative immunoblot of the Syntenin expression in WT and KO cells and sEVs, including HSP90 as a housekeeper, is shown on the left. (B and C) MS: Principal component analysis (B) of WT and Syntenin KO sEVs. Circles indicate similarity clusters. Volcano plot (C) with significantly differentially expressed proteins (*p* < 0.05). (D) Pathway enrichment analysis for all 178 sEV proteins significantly downregulated (*p* < 0.05) upon Syntenin KO. Shown are the top three associations based on Gene Ontology (GO) (cellular component [CC]) and Kyoto Encyclopaedia of Genes and Genomes (KEGG) databases. Adhesion‐related processes are highlighted in orange. The numbers given in the individual bars indicate the number of proteins identified in the MS analysis in relation to the total number of proteins in the respective pathway. (E) The significantly downregulated proteins were grouped according to their molecular function. Cell adhesion proteins were further divided into proteins belonging to the ECM, mediating extracellular cell–cell/cell‐ECM interactions or intracellular adhesion‐related cytoskeletal rearrangements. Proteins selected for validation were marked in bold.

Due to the known function of Syntenin as a key regulator of exosome biogenesis as well as its high enrichment on sEVs (Figure S1D), we concentrated our study on this EV subpopulation. A CRISPR/Cas9‐mediated knockout (KO) was introduced in 4T1 cells, and Syntenin loss‐of‐function was validated in two independent KO clones (KO_1, KO_2) compared to wild‐type (WT) cells by immunoblotting of corresponding cell lysates and sEV samples (Figure 1A). To assess the influence of Syntenin depletion on the total proteome of the sEVs, we characterised them by label‐free tandem MS. The analysis yielded a total number of 1251 detected proteins. According to the MISEV 2023 categories for protein‐based EV characterisation (Welsh et al. 2024), we detected EV‐associated proteins linked to (i) endosomal or plasma membranes (tetraspanins: CD9, CD63, CD81; epithelial marker: EpCAM), (ii) the cytosol (ESCRT proteins: CHMP1‐6, TSG101; heat shock proteins: HSPA8, HSP70), and (iii) secretion (ECM proteins: FN1, several collagens) (Figure S1E). Furthermore, as reported by others (Lázaro‐Ibáñez et al. 2019), we identified several DNA‐binding proteins (i.e. histones) on sEVs. Next to Albumin as the only serum‐associated contaminant, we detected four 14‐3‐3 proteins (YWHAB, YWHAQ, YWHAZ, SFN), which are generally more abundant on extracellular particles than on EVs (Welsh et al. 2024).

Principal component analysis demonstrated a high level of similarity between the sEV proteomes of both Syntenin KO clones, with protein profiles distinctly different compared to those from 4T1 WT sEVs (Figure 1B,C). Of note, the replicates of the KO sEV samples clustered more tightly together than the replicates of the WT sEVs. This suggests that the loss of Syntenin restricts the range of proteins sorted into sEVs, resulting in a more homogenised proteomic profile compared to WT sEVs. Such a finding would be consistent with the idea that Syntenin plays a broad regulatory role in sEV cargo selection, leading to more limited cargo sorting mechanisms in its absence. Out of the 1251 detected proteins on sEVs, 414 showed a significantly (*p* < 0.05) different expression upon Syntenin depletion, including 178 downregulated and 236 upregulated proteins (Table S2). The set of sEV proteins with reduced expression comprised known Syntenin binding partners like SDC4, ALIX, CD63, and SRC (Figure S2A). Moreover, pathway enrichment analysis based on the Gene Ontology (GO) (cellular component [CC]) and the Kyoto Encyclopedia of Genes and Genomes (KEGG) database revealed that many of the downregulated proteins were associated with adhesion‐related processes (Figure 1D). The top three associations according to GO (CC) were ‘focal adhesion’, ‘cell‐substrate junction’ and ‘vesicle’. Consistently, two of the most significantly regulated pathways according to KEGG were ‘ECM‐receptor interaction’ and ‘regulation of actin cytoskeleton’. Indeed, when assigning the 178 downregulated proteins to their main molecular function, the majority of them (*n* = 56) were associated with cell adhesion, followed by EV biogenesis (*n* = 39) and metabolism (*n* = 34) (Figure 1E), thus confirming the results of the pathway enrichment analysis. Noteworthy, cell adhesion seemed to be exclusively linked to the set of sEV proteins down‐regulated upon Syntenin KO, since the 236 sEV proteins with significantly increased expression were mostly associated with ribosomal pathways (Figure S2B). In summary, our data revealed that Syntenin loss‐of‐function has a major impact on the proteome of sEVs in breast cancer cells, in particular sEV cargo proteins associated with adhesion.

### 3.3 Syntenin Regulates the Expression of Adhesion Proteins on sEVs

**FIGURE 2:**
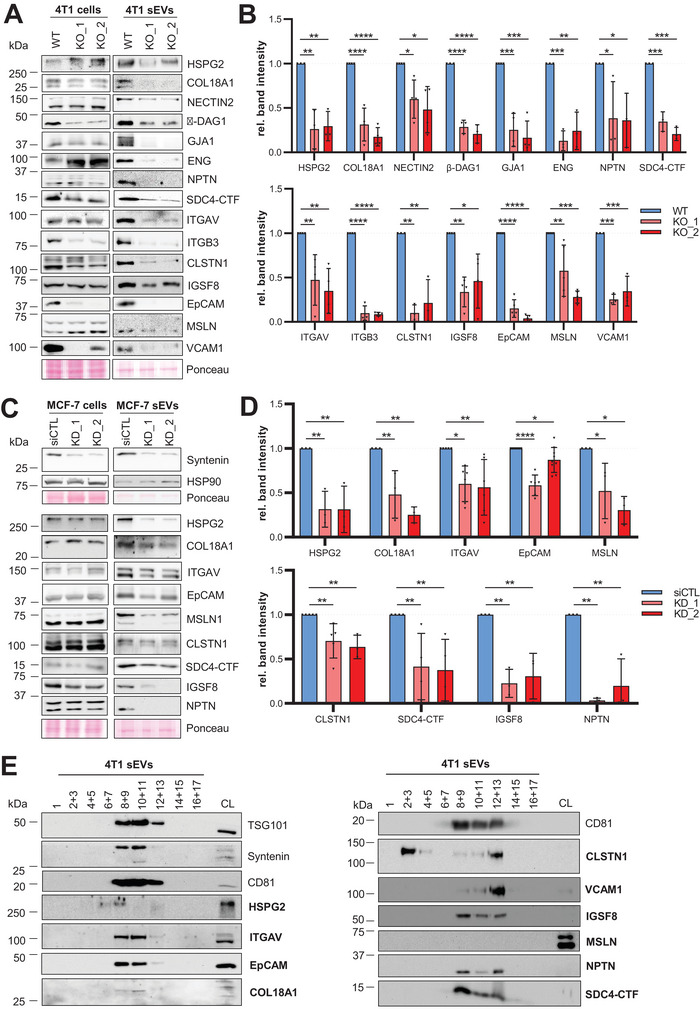
Syntenin regulates the expression of adhesion proteins on breast cancer sEVs. (A–D) Immunoblots: Expression of selected adhesion proteins in cell lysates and sEVs from 4T1 wild‐type (WT) and Syntenin knockout (KO_1/2: 2 clones) cells (A) or from MCF‐7 cells treated with either control siRNA (siCTL) or an siRNA directed against Syntenin (KD_1/2: 2 siRNA sequences) (C). The same amount of protein was loaded for all cell lysates and for all sEVs. Protein expression on sEVs was quantified by densitometry (B and D) and is shown in relation to the WT, or siCTL control, respectively. (E) 4T1 WT sEVs isolated by ultracentrifugation were additionally purified on an Optiprep density gradient. The expression of the validated adhesion proteins in the different fractions was analysed by immunoblotting. TSG101, Syntenin and/or CD81 served as sEV markers. The 4T1 WT input cell lysate (CL) is shown as a positive control.

To further investigate the signature of Syntenin‐dependent adhesion proteins on sEVs, we selected all proteins for further validation that fulfilled the following two criteria: (i) Transmembrane or extracellular expression, (ii) core ECM protein or protein involved in direct cell–cell or cell‐matrix interactions. The resulting 26 proteins (marked in bold in Figure 1E) were analysed by immunoblotting on 4T1 WT and Syntenin KO cells and sEVs. As the stable Syntenin loss‐of‐function had no effect on the number of secreted sEVs or the EV particle/protein ratio (Figure S3A,B), we chose to load the same amount of EV protein in all conditions for the comparative analysis. The immunoblots confirmed a measurable downregulation for 15/26 cell adhesion‐associated proteins on Syntenin‐depleted sEVs (Figure 2A,B, Figure S3C,D). CD47 was only detectable in considerable amounts in the cell lysate but not on the corresponding sEVs. Of note, sEV‐associated DAG1 deficiency was only observed for the membrane‐associated subunit β‐DAG1, whereas extracellular α‐DAG1 levels remained unchanged upon Syntenin KO. For five of the downregulated sEV proteins (CLSTN1, ITGB3, β‐DAG1, EpCAM, VCAM1), we measured decreased protein levels in corresponding Syntenin KO cell lysates (Figure S3E). Hence, it cannot be ruled out that for these proteins, their loss on Syntenin KO sEVs arises from non‐EV‐related cellular effects of Syntenin.

Next, we asked whether the observed Syntenin‐mediated regulation of the adhesion proteins on sEVs also applies to human breast cancer cells. To this end, Syntenin was transiently silenced in MCF‐7 cells using two distinct siRNAs (KD_1, KD_2) (Figure 2C) and the differential expression of the validated 15 adhesion proteins was investigated on sEVs and corresponding cell lysates compared to control sEVs from cells transfected with a non‐targeting siRNA (siCTL). In contrast to the stable KO, transient reduction of Syntenin expression significantly decreased the number of secreted sEVs (Figure S4A), consistent with previous reports (Baietti et al. 2012). Since the ratio of particles per µg EV protein was comparable between siCTL and Syntenin KD sEVs (Figure S4B), we again loaded the same amount of EV protein per lane on the immunoblots for quantitative comparison of EV protein expression. We observed a consistent Syntenin‐dependent downregulation of nine of the proteins in the MCF‐7 cells, including HSPG2/Perlecan, COL18A1, ITGAV/Integrin alpha‐V, EpCAM, MSLN/Mesothelin, CLSTN1/Calsyntenin‐1, IGSF8, NPTN/Neuroplastin and SDC4 C‐terminal fragments (CTFs) (Figure 2C,D). ITGB3/Integrin beta‐3 and β‐DAG1 only showed decreased expression levels on sEVs for one of the two siRNAs, while NECTIN2, ENG/Endoglin and GJA1/Connexin‐43 remained unchanged (Figure S4C,D). VCAM1 was only barely detectable in MCF‐7 cells and not present on their sEVs (Figure 2C). In contrast to 4T1 cells, Syntenin loss‐of‐function had no considerable effects on the levels of any of the proteins in MCF‐7 cell lysates (Figure S4E).

Since EV isolation by differential ultracentrifugation might lead to the co‐isolation of protein aggregates and lipid particles, we further purified the EVs from 4T1 cells on Optiprep density gradients to confirm the association of the validated adhesion proteins with sEVs (Figure 2E). Indeed, we found all of them to be associated with fraction 8 to 13 which we had previously identified as EV fractions in this setting (Schöne et al. 2024). Only MSLN did not produce any signals in the density gradient, while CLSTN1 was also detectable in fraction 2–5 hinting at its additional association with non‐EV particles. Moreover, CLSTN1 and VCAM1 showed a particular enrichment in fraction 12–13, whereas the other proteins showed stronger signals in fraction 8–11 which could point to their expression on distinct sEV subpopulations. Taken together, our analyses confirmed Syntenin as a conserved regulator for the expression of a selected set of adhesion proteins on sEVs in breast cancer.

### 3.4 Syntenin Regulates the Adhesive Capacity of sEVs

**FIGURE 3:**
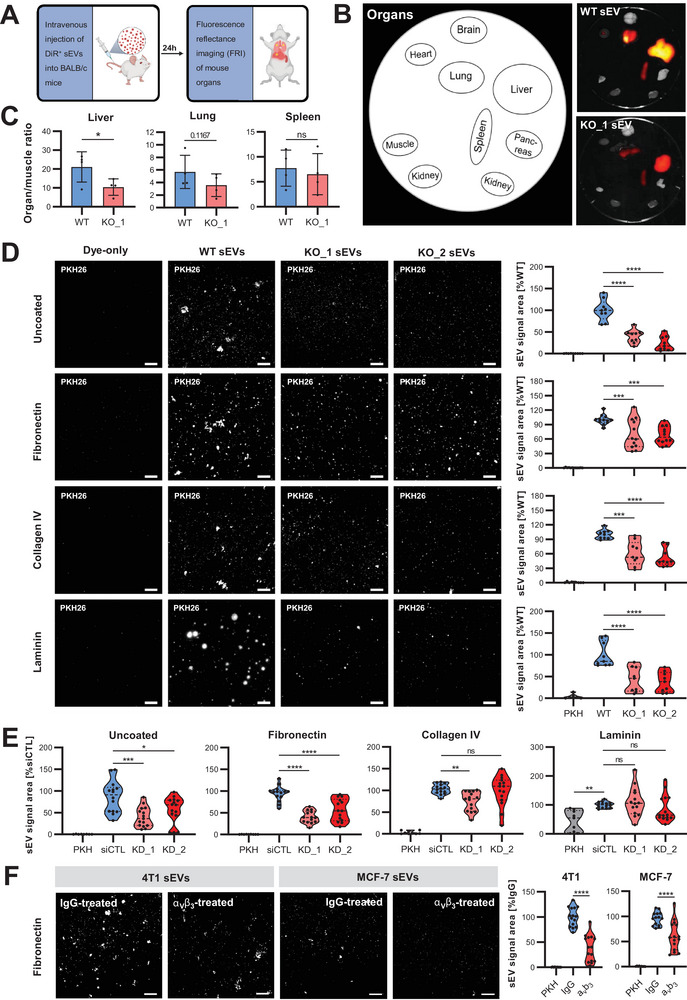
Syntenin regulates the adhesion of sEVs in vivo and in vitro. (A–C) Biodistribution of 4T1 sEVs after tail vein injection in a syngeneic mouse model. Shown is the workflow (A) and representative images of the DiR fluorescence in the main organs 24 h post‐injection as measured by ex vivo fluorescence reflectance imaging (FRI) (B). For the subtraction of background signals, raw intensity values for each organ were divided by auto‐fluorescent muscle signals. Organ/muscle ratio differences between WT and Syntenin KO sEVs (C) were tested for liver, lung and spleen using a one‐sided *t*‐test (*p < 0.05; ns: not significant). (D) Confocal microscopy: PKH26‐labelled sEVs from 4T1 WT and Syntenin KO (KO_1/2: 2 clones) were seeded on glass coverslips coated with different ECM components. Scale bar: 20 µm. Shown are representative images (left) and the corresponding quantification of thresholded PKH26 signal areas (right). (E) PKH26‐labelled sEVs from MCF‐7 treated with control (siCTL) and Syntenin siRNA (KD_1/2: 2 sequences) were incubated on glass coverslips coated with different ECM components, and thresholded PKH26 signals of adherent sEVs were quantified. (F) 4T1 and MCF‐7 sEVs pre‐treated with a blocking antibody against Integrin‐αVβ3 or a corresponding IgG control (20 µg/mL) were seeded on Fibronectin‐coated glass coverslips and visualised by confocal imaging. Thresholded PKH26‐signal areas were normalised to IgG‐treated sEV signals. Shown are representative images (left) and the corresponding quantification of thresholded PKH26 signal areas (right).

Adhesion proteins on tumour‐derived sEVs, especially in the case of Integrins, are known to influence sEV organotropism and thus metastatic niche formation (Hoshino et al. 2015). Hence, we aimed to investigate whether the loss of vesicular adhesion proteins upon Syntenin loss‐of‐function would influence the biodistribution of sEVs. For this analysis, we took advantage of the previously described syngeneic mouse model (Gerwing et al. 2020) and intravenously injected DiR‐labelled sEVs from 4T1 WT and Syntenin KO cells into the tail vein of female BALB/c mice (Figure 3A). We refrained from including dye‐only control injections due to results from our previous work, which had demonstrated the absence of unspecific DiR‐signals in stained sEV preparations (Irmer et al. 2023b). Fluorescent signals of DiR^+^ sEVs were measured 24 h after injection by ex vivo FRI of the different organs. The analysis detected fluorescent signals of the injected sEVs in the lungs, spleen and liver, which were markedly decreased in the latter upon Syntenin loss‐of‐function (Figure 3B,C). There was also a trend towards decreased homing of the tumour sEVs to the lungs; however, it did not reach statistical significance. No such effect was observed for sEV signals in the spleen.

Encouraged by these results, we next aimed to investigate the binding of sEVs to ECM proteins representing potential targets for sEV‐associated Integrins and other adhesion proteins. Strikingly, PKH26‐labelled sEVs from 4T1 Syntenin KO cells were less adhesive to Fibronectin, Collagen IV and Laminin compared to WT sEVs as shown by confocal microscopy (Figure 3D). Accordingly, sEVs from MCF‐7 Syntenin KD cells exhibited decreased binding to Fibronectin‐coated and uncoated coverslips (Figure 3E, Figure S5A). However, signal areas of sEVs adherent to Collagen IV and Laminin were not considerably altered upon Syntenin silencing in MCF‐7, pointing towards Fibronectin as a conserved target for Syntenin sEVs. Based on our data, which had confirmed reduced levels of the Fibronectin binding partners ITGAV and ITGB3 on Syntenin‐depleted sEVs, we tested Integrin alpha‐V/beta‐3 (α_V_β_3_) as a potential mediator for the reduced Fibronectin binding capacity. Indeed, pre‐incubation with a blocking antibody against α_V_β_3_ significantly decreased the binding of sEVs from 4T1 and MCF‐7 cells to Fibronectin (Figure 3F). These results suggest that Syntenin regulates the adhesion of sEVs to Fibronectin by directing α_V_β_3_ to sEV membranes. Of note, we detected the two Integrin‐activating proteins, Talin‐1 and Kindlin‐2, on sEVs from both cell lines (Figure S5B). As their expression did not change upon Syntenin loss‐of‐function, we assume sEV‐associated Integrins to be functional receptors for ECM proteins and their Syntenin‐mediated expression on sEVs to be responsible for the regulation of vesicular Fibronectin binding.

### 3.5 Breast Cancer Cells Show Enhanced Directional Cell Movement on Syntenin sEVs

**FIGURE 4:**
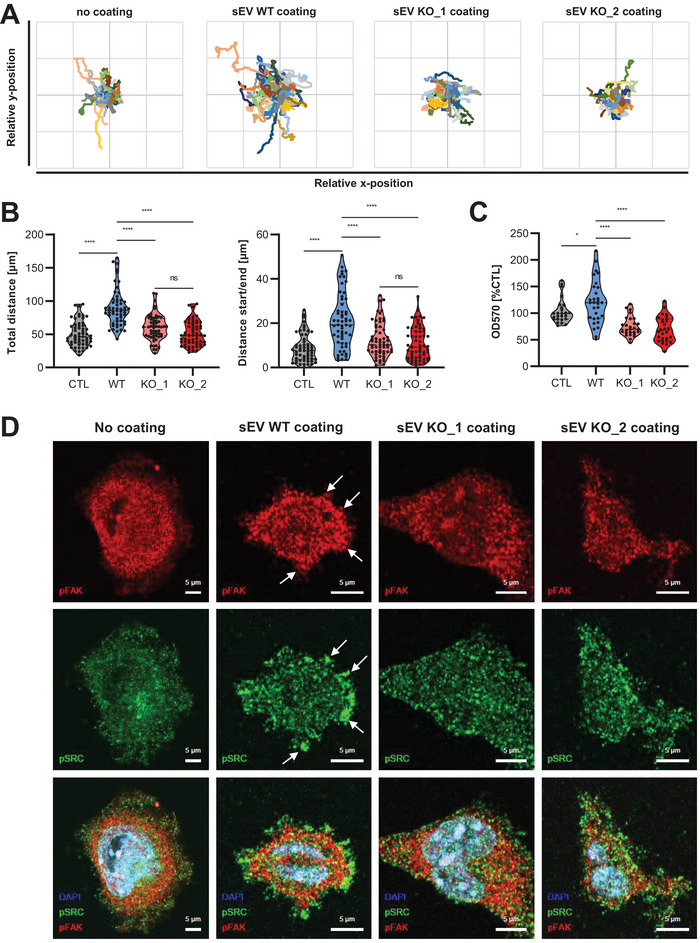
Syntenin sEVs stimulate the movement of 4T1 breast cancer cells. (A and B) 4T1 cells were seeded on either PBS (CTL), 4T1 WT or Syntenin KO (KO_1/2: 2 clones) sEVs and tracked for 8 h by live cell imaging. Based on the manually tracked migration of individual cells (A), the total migration distance as well as the directional cell movement were calculated (B). Violin plots indicate the median, lower and upper quartiles (*n* = 60). (C) 4T1 cells were cultured for 10 min on wells coated with either PBS (CTL), WT, or Syntenin KO sEVs and cell adhesion was quantified by crystal violet staining. Violin plots display the median, lower and upper quartiles (*n* = 30). (D) Confocal imaging: 4T1 cells were seeded on WT or Syntenin KO sEVs and stained for phosphorylated FAK (pFAK) and SRC (pSRC) as markers for Integrin downstream signalling. White arrows point to some exemplary signals enriched at cell protrusions. Scale bar: 5 µm.

Cancer cells show enhanced directional movement upon binding to sEVs, which trigger the formation of adhesion assemblies in an Integrin‐dependent manner (Sung et al. 2015). We therefore asked whether Syntenin loss‐of‐function would affect this sEV function. To address this question, 4T1 cells were seeded on top of WT and Syntenin KO sEVs, and single‐cell migration was analysed by live cell imaging. Confirming previous data, 4T1 cells showed a significant increase in their total migrated distance as well as in their directional cell movement on top of WT sEVs compared to the uncoated control. In contrast, Syntenin KO sEVs failed to induce tumour cell migration (Figure 4A,B). To test the hypothesis that this effect was caused by a weakened adhesion of the cells to Syntenin KO sEVs, we measured the amount of adherent cells after short‐term incubation on WT or Syntenin KO sEVs by crystal violet staining. The analysis confirmed that 4T1 cells indeed displayed a stronger adhesion to WT than to Syntenin KO sEVs (Figure 4C). In line, 4T1 cells showed an enhanced expression of phosphorylated, and thus active, FAK and SRC at peripheral cell protrusions only on pre‐coated WT sEVs as visualised by confocal imaging (Figure 4D).

Upon incubation of MCF‐7 cells on top of siCTL sEVs, we only observed an induction of directional cell movement, but not of total migration distance or adhesion to sEVs (Figure S6A–C). Likewise, no significant differences were observed for the migration‐ or adhesion‐promoting capacity of Syntenin KD sEVs (Figure S6A–C). Whether these differences arise from cell line‐specific effects, the only partial Syntenin KD in MCF‐7 compared to the complete KO in 4T1, or from the more pronounced expression differences of the adhesion proteins on sEVs from the latter, remains to be tested. Taken together, these data suggested that Syntenin‐negative sEVs are less potent stimulators for Integrin activation, and thus cell migration and adhesion, at least in the 4T1 model.

### 3.6 Syntenin sEVs Stimulate Cell Migration Despite Inhibition of EV Uptake

**FIGURE 5:**
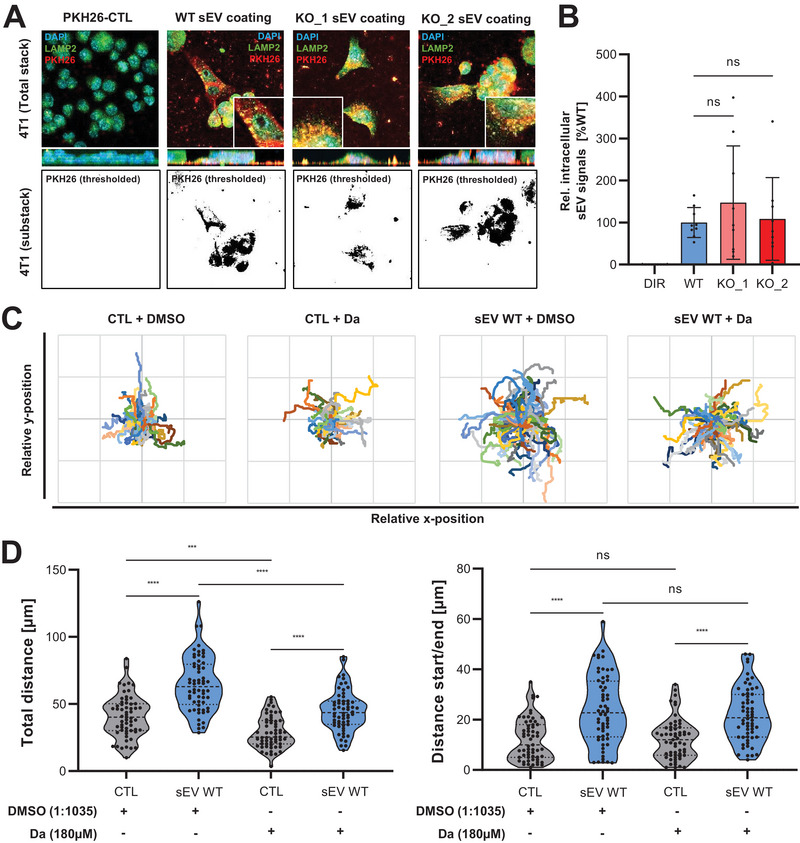
Syntenin KO sEVs are taken up into 4T1 cells and induce breast cancer cell migration despite inhibition of EV uptake. (A and B) Confocal microscopy: 4T1 cells were seeded on top of PKH26‐labelled WT or Syntenin KO (KO_1/2: 2 clones) sEVs and stained for the late endosomal marker LAMP2 (A). Depicted are maximal intensity summations of total Z‐stacks with a 90° angle side‐view on the x‐axis (upper pictures) and thresholded substacks representing intracellular PKH26‐signals (lower pictures). Intracellular sEV signal areas were normalised to the total cell area per picture (B). (C and D) 4T1 cells pre‐treated for 2 h with DMSO or Dansylcadaverine (180 µM) were seeded on WT sEVs and tracked for 8 h by live‐cell imaging. Based on the manually tracked migration of individual cells (C), the total migration distance and the directional cell movement were calculated (D). Violin plots display the median, lower and upper quartiles (*n* = 60).

In order to investigate whether the effect of sEVs on cell adhesion was solely caused by cell‐EV interactions or by the transfer of adhesion‐promoting cargo molecules, we analysed sEV uptake into 4T1 cells. Confocal microscopy of 4T1 cells seeded on top of PKH26‐labelled 4T1 sEVs elucidated the cellular uptake of the vesicles as represented by a co‐localisation of the sEV signals with the late endosomal marker LAMP2 (Figure 5A). Quantifying the intracellular levels of WT and Syntenin KO sEVs did not detect significant differences, suggesting that EV uptake was not affected by Syntenin loss‐of‐function (Figure 5A,B, Figure S7). To test the relevance of EV uptake for the pro‐migratory function of the sEVs, we first aimed to identify an inhibitor that would allow us to efficiently block the incorporation of sEVs into 4T1 cells. To this end, 4T1 were pre‐treated for 2 h with several known inhibitors of EV uptake, including dynasore, dansylcadaverine, filipin and simvastatin, which block different endocytic pathways (Mulcahy et al. 2014), prior to the addition of DiR‐labelled sEVs. Flow cytometry‐based quantification of EV uptake 24 h post‐stimulation revealed that only dansylcadaverine was able to efficiently block vesicle incorporation into 4T1 by 50% at a concentration that was non‐toxic for the cells (Figure S8A,B). Despite the inhibition of EV uptake, dansylcadaverine failed to antagonise the sEV‐induced increase of 4T1 cell migration, both for the total migrated distance as well as the directional cell movement (Figure 5C,D). In summary, these observations propose that the pro‐migratory effect of Syntenin sEVs from 4T1 cells is independent of EV uptake and seems to be rather mediated by focal adhesions between the cells and the underlying sEVs.

## 4 Discussion

**FIGURE 6:**
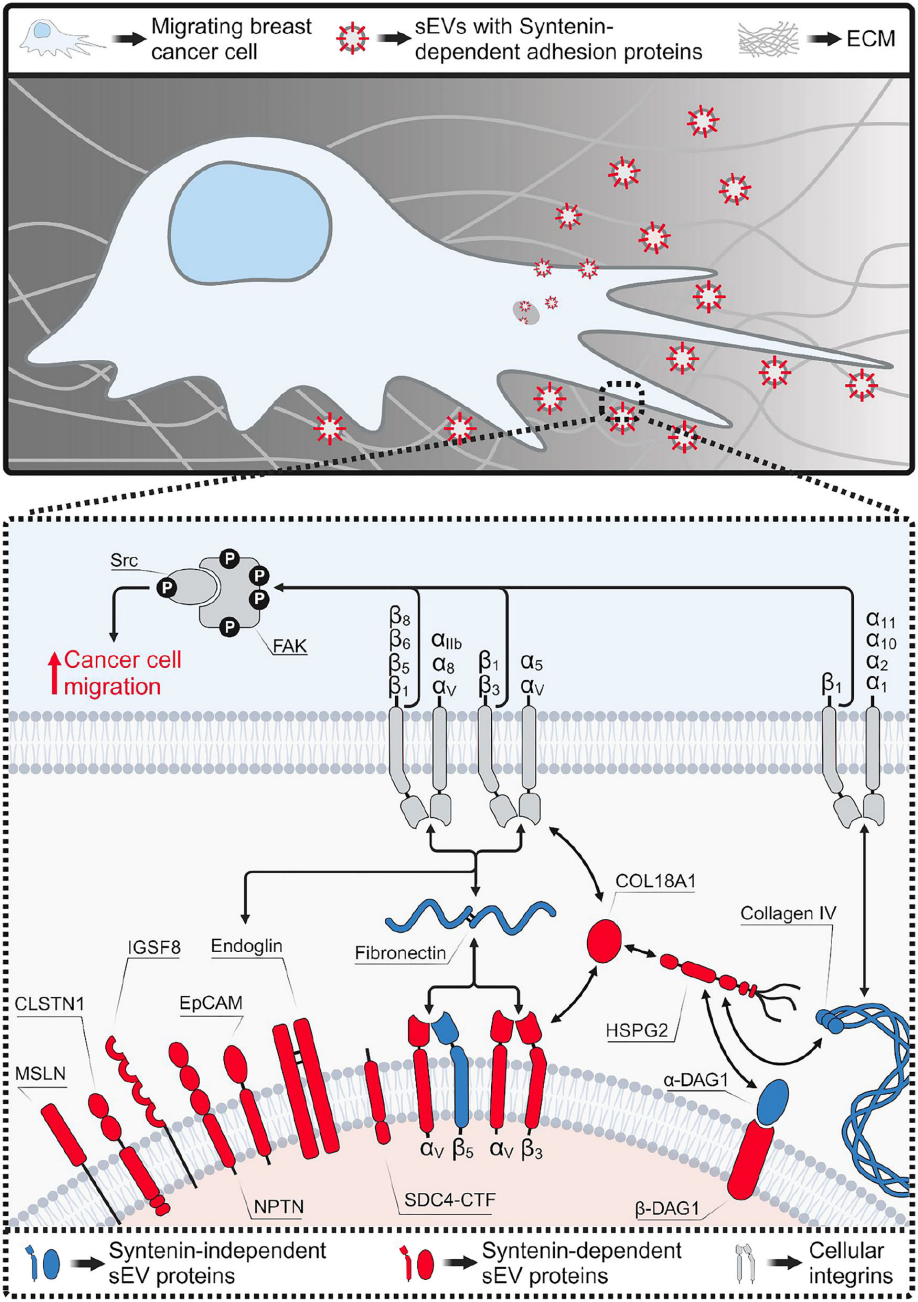
Model of Syntenin regulating sEV‐induced breast cancer cell adhesion and migration. Syntenin was found to regulate the expression of adhesion proteins (red) on sEVs. On the one hand, these proteins can act as anchors to mediate sEV adhesion to the ECM, in particular to Fibronectin. On the other hand, they can serve as tethers to promote Integrin‐dependent breast cancer cell adhesion and migration. The figure was created in BioRender. Menck, K. (2025) https://BioRender.com/l01h906.

A growing number of studies have revealed a considerable amount of heterogeneity among the sEV population, which comprises not only exosomes, but also EVs originating from the plasma membrane as well as non‐vesicular particles (i.e. suprameres and exomeres) (Jeppesen et al. 2023). In this work, we focused on Syntenin‐dependent sEVs, presumably reflecting endosomal‐derived exosomes, to characterise their proteomic cargo and relate it to the tumour‐supportive role of Syntenin sEVs in breast cancer. We found that Syntenin was responsible for regulating a large number of adhesion proteins on sEVs. Syntenin loss‐of‐function was associated with a markedly diminished adhesive potential of the sEVs in vitro and in vivo. As a consequence, Syntenin‐negative sEVs failed to induce breast cancer cell migration, thereby identifying Syntenin as an important regulator of a tumour‐supportive sEV phenotype (Figure 6).

Within the last decade, EV subtyping has become increasingly reliant on the attribution of vesicles to certain proteomic signatures, which do not necessarily reflect distinct routes for EV biogenesis but rather certain states and properties of the cellular origin. In this regard, Syntenin‐dependent sEVs, analysed in this work, might be enriched for, but not exclusive to, exosomes, and rather represent a heterogeneous subpopulation with a distinct proteomic profile and tumour‐supporting potential. The majority of the adhesion proteins were exclusively regulated on sEV membranes, suggesting that the function of Syntenin is attributable to an effect on sEV cargo loading. PDZ binding motifs have been reported for GJA1 (Giepmans and Moolenaar 1998), NECTIN2 (Duraivelan and Samanta 2020), VCAM1 (van Buul and Hordijk 2009) and EpCAM (Leblanc et al. 2020) and could thus mediate a direct interaction with either of the two PDZ domains of Syntenin to regulate their export onto sEVs as it has been described for the CTFs of SDCs (Grootjans et al. 1997; Baietti et al. 2012). In contrast, the Syntenin‐dependent export of immunoglobulin superfamily members (i.e. NPTN, IGSF8) or Integrins onto sEVs could be mediated via PDZ‐binding tetraspanins. Indeed, CD63 and CD9, both established Syntenin binding partners with reduced expression levels in Syntenin KO sEVs, have been reported to form complexes with IGSF8 (Charrin et al. 2003), ITGAV (Chang and Finnemann 2007; Zhang et al. 2022) and ITGB3 (Israels et al. 2001). Similarly, the observed differential expression of sEV‐associated HSPG2 and COL18A1 could potentially be mediated by the Syntenin‐dependent expression of corresponding binding partners: HSPG2 is known to interact with DAG1 and Fibronectin (Guilak et al. 2021), which in turn, like COL18A1, is a ligand for Integrin‐α_V_β_3_ (Plow et al. 2000; Faye et al. 2009). Decreased levels of HSPG2, β‐DAG1 and Integrin‐α_V_β_3_ could explain the deficient binding of Syntenin KO sEVs to Fibronectin, Collagen IV and Laminin: HSPG2 interacts with all three ECM components analysed (Guilak et al. 2021), DAG1 is a known receptor for Laminin (Millar et al. 1989) and Integrin‐α_V_β_3_ specifically binds Fibronectin (Plow et al. 2000). Although SDC4 can equally bind Fibronectin and Laminin, this function depends on its extracellular domain (Mahalingam et al. 2007; Carulli et al. 2012) which is cleaved by heparanase during exosome formation (Roucourt et al. 2015), leaving mostly SDC‐CTFs on the vesicles. Still, SDC4‐CTFs can activate PKCα, which, together with Talin, is important for Integrin activation (Morgan et al. 2007) and could thus contribute to the Fibronectin‐binding capacity of the sEVs.

Defective Fibronectin binding was conserved between Syntenin‐depleted sEVs from 4T1 and MCF‐7 cells and seemingly caused by the reduced Integrin‐α_V_β_3_ levels on the respective sEVs. In 2015, Hoshino et al. demonstrated that Integrins expressed on the surface of tumour‐derived sEVs determine organ‐specific EV uptake and metastasis formation (Hoshino et al. 2015). In particular, sEV‐associated ITGAV was shown to contribute to the infiltration of the Fibronectin‐enriched liver microenvironment upon dimerisation with ITGB5 and additionally served as a prognostic marker for liver metastasis in pancreatic cancer patients. Consistently, we observed reduced hepatic uptake of ITGAV‐deficient Syntenin KO sEVs with an evidently reduced Fibronectin binding affinity and, hence, Syntenin as a potential regulator for the Integrin‐dependent liver tropism of tumour‐derived sEVs. How far this distribution effect depends on Integrin‐α_V_β_3_ or other ITGAV‐containing Integrins under the control of Syntenin, like for example, Integrin alpha‐V/beta‐5 (α_V_β_5_), remains to be elucidated. A decreased dimerisation of ITGAV with ITGB6 or ITGB8 on Syntenin‐deficient sEVs as a cause for reduced Fibronectin binding can be excluded, since, based on the proteomic data, both proteins were found to be absent on sEVs from 4T1 cells. The heterodimerisation of Integrins is only the first step for the proper formation of focal adhesions to ECM proteins. Conformational changes of Integrin dimers from a ‘bent closed’ to an ‘extended closed’ state upon C‐terminal binding of Talin and Kindlin, which were shown to be inevitable for their function as ECM receptors (Lu et al. 2022). Unlike previous studies about functional implications of sEV‐associated Integrins, we detected both Talin‐1 and Kindlin‐2 on 4T1‐derived sEVs, suggesting a functional inside‐out activation of Integrins on sEVs.

Next to ITGAV, we observed eight other cell adhesion proteins to be controlled by Syntenin for their export onto sEVs from 4T1 and MCF‐7 cells. This conserved effect indicates Syntenin generally regulates adhesion proteins on sEVs in breast cancer, not exclusive to one species or cancer subtype. Still, compared to the murine 4T1 cell line, sEVs from Syntenin‐silenced human MCF‐7 cells displayed less differentially expressed adhesion proteins. In addition, we only detected a mild effect on directional cell migration, but not total migration distance, pointing to a lesser pro‐migratory function of sEVs from this cell line per se. Next to the expression of residual Syntenin molecules during transient RNAi‐mediated knockdown, these differences might be a consequence of cancer subtype‐specific Syntenin expression and function. Previous studies reported Syntenin to be specifically overexpressed in triple‐negative breast cancer (TNBC) compared to hormone receptor‐positive cell lines of the Luminal A and Luminal B subtypes. (Koo et al. 2002; Qian et al. 2013). Furthermore, high Syntenin expression in TNBC was linked to increased proliferation and migration, whereas estrogen receptor signalling counteracted Syntenin expression in Luminal A breast cancer cells (Koo et al. 2002; Qian et al. 2013). Accordingly, the observed differences between triple‐negative 4T1 and hormone receptor‐positive MCF‐7 cells regarding the impact of Syntenin depletion on sEV composition and function appear reasonable and should be addressed in comparative studies including human TNBC cell lines such as MDA‐MB‐231 (González‐King et al. 2022).

In line with the high tumourigenic potential of Syntenin in TNBC, we identified a regulatory function of Syntenin on the pro‐migratory capacity of sEVs exclusively in 4T1 cells. As initially discovered in fibrosarcoma cells, tumour‐derived sEVs can function as an adhesive platform for inducing cancer cell migration (Sung et al. 2015). Concomitantly, Integrin‐bound Fibronectin was identified as an important sEV cargo for the binding of migrating cancer cells via focal adhesions. However, the depletion of Fibronectin on sEVs alone was shown to be insufficient for blocking sEV‐mediated directional cell movement (Sung and Weaver 2017), suggesting the presence of other migration‐promoting sEV cargo molecules. Indeed, in our study, we did not observe an effect of Syntenin depletion on Fibronectin expression on sEVs. Instead, in our model system, the Syntenin‐regulated export of Endoglin to sEVs could represent an alternative mechanism: Beyond its function as a co‐receptor for TGF‐β signalling (Rossi et al. 2019), Endoglin on the surface of endothelial cells has been shown to facilitate adhesion to leukocytes (Rossi et al. 2013), platelets (Rossi et al. 2017) and mural cells (Rossi et al. 2016) via its extracellular RGD‐motif, a binding platform for Fibronectin‐binding Integrins. In a soluble form, however, Endoglin was found to block endothelial Integrins, thereby supporting podocyte detachment (Rossi et al. 2016). Hence, sEV‐associated Endoglin might be a target for the Integrins of migrating breast cancer cells, concomitantly supporting epithelial detachment as a competitive inhibitor of intercellular junctions. Alternatively, Syntenin could potentially regulate the formation of RGD‐independent focal adhesions between cellular Integrins and sEV proteins by controlling Collagen IV and its binding partner HSPG2 on sEVs. Furthermore, Syntenin might regulate interactions between the cell and the sEV membrane by controlling the vesicular expression of CLSTN1, IGSF8, NPTN, NECTIN2, EpCAM or GJA1 which can form either homo‐ or heterophilic interactions with cognate receptors on the cell surface with a yet unknown impact on cell migration.

In previous studies, Integrin‐α_V_β_3_ on the surface of prostate cancer‐derived sEVs was shown to be transferred to target cell membranes, thereby activating a migratory phenotype in recipient cells (Singh et al. 2016; Krishn et al. 2019). Hence, we tested the dependence of sEV‐induced cancer cell migration on EV uptake. Our data, however, argue for the hypothesis that cancer cell motility on sEVs predominantly relies on adhesive interactions between cells and vesicles independent of EV uptake. Furthermore, despite reports about sEV‐associated Integrin‐α_V_β_3_ as a regulator for EV incorporation into recipient breast cancer cells (Altei et al. 2020), we observed no decrease in the uptake of Integrin‐α_V_β_3_‐deficient Syntenin KO sEVs into above‐seeded cancer cells. Hence, the loss of the pro‐migratory capacity of Syntenin KO sEVs cannot be attributed to decreased uptake into recipient breast cancer cells. How far the uptake of other Syntenin‐dependent tumour molecules on sEVs, like for example, SRC, impacts downstream signalling, and thus also the migration of target cells, still needs further investigation.

In summary, we show for the first time that Syntenin, in line with its established role as a key regulator of exosome biogenesis, is responsible for controlling the proteomic cargo of sEVs in breast cancer on a large scale. This Syntenin‐dependent tumour sEV subpopulation with an adhesive protein signature was linked to liver tropism and enhanced cancer cell migration, potentially mediated by sEV‐associated focal adhesion proteins under the control of Syntenin. Taken together, these findings pave the way for the discovery of sEV‐associated biomarkers for Syntenin‐overexpressing cancers and may help to unravel molecular mechanisms behind specific pathological functions of tumour‐derived sEVs. Considering the recent identification of a Syntenin‐specific inhibitor (Leblanc et al. 2020), our results further suggest that targeting Syntenin could represent a promising therapeutic strategy to counteract breast cancer metastasis, warranting further investigation in preclinical and clinical settings.

## Author Contributions

**Barnabas Irmer**: Conceptualization (equal); formal analysis (equal); investigation (lead); visualization (lead); writing–original draft (equal); writing–review and editing (equal). **Allegra Angenendt**: Investigation (supporting); writing–review and editing (equal). **Luc Camoin**: Formal analysis (equal); investigation (supporting); methodology (equal); writing–review and editing (equal). **Stéphane Audebert**: Investigation (supporting); methodology (equal); writing–review and editing (equal). **Christiane Geyer**: Investigation (supporting); writing–review and editing (equal). **Mirjam Gerwing**: Methodology (equal); writing–review and editing (equal). **Hanna Spiessbach**: Investigation (supporting); writing–review and editing (equal). **Mira Hebel**: Investigation (supporting); methodology (supporting); writing–review and editing (equal). **Émilie Baudelet**: Investigation (supporting); writing–review and editing (equal). **Darius Wlochowitz**: Formal analysis (supporting); writing–review and editing (equal). **Uwe Hansen**: Investigation (supporting); methodology (supporting); writing–review and editing (equal). **Annalen Bleckmann**: Resources (equal); writing–review and editing (equal). **Pascale Zimmermann**: Conceptualization (supporting); funding acquisition (supporting); resources (equal); writing–review and editing (equal). **Kerstin Menck**: Conceptualization (equal); funding acquisition (lead); investigation (supporting); project administration (lead); supervision (lead); validation (lead); writing–original draft (equal); writing–review and editing (equal).

## Ethics Statement

All animal experiments in this study have been approved by the responsible local authorities (approval 81‐02.04.2021.A397). All applicable institutional and/or national guidelines for the care and use of animals were followed.

## Conflicts of Interest

The authors declare no conflicts of interest.

## Supporting information




**Supplementary Figures and Table**: jev270133‐sup‐0001‐SuppMat.pdf

**Supporting Table 1**: jev270133‐sup‐0001‐tableS2.xlsx

## Data Availability

All data supporting the findings of this study are available within the article and the supplemental information, or from the corresponding author upon reasonable request. The MS data have been deposited in the ProteomeXchange Consortium via the PRIDE partner repository with the dataset identifier PXD007721 (https://www.ebi.ac.uk/pride/).
