# Supplementary material for: Syntenin Controls Extracellular Vesicle‐Induced Tumour Migration by Regulating the Expression of Adhesion Proteins on Small Extracellular Vesicles
Source: J Extracell Vesicles. 2025 Aug 20;14(8):e70133. doi: 10.1002/jev2.70133 (PMC12365386; doi:10.1002/jev2.70133)
Supplement: Supplementary file 1 — Supplementary Figures and Table: jev270133‐sup‐0001‐SuppMat.pdf [file JEV2-14-e70133-s001.pdf]

Suppl. Table 1: Antibody details.

Gene	Protein	Phospho Specificity	Supplier	Antibody ID	Host	Dilution
ACTB	β-Actin		santa cruz	#sc-47778	mouse	1:2000
ARF6	ADP-ribosylation factor 6		santa cruz	#sc-7971	mouse	1:1000
CD47	Leukocyte surface antigen CD47		santa cruz	#sc-12730	mouse	1:250
CD81	CD81 antigen		santa cruz	#sc-166028	mouse	1:1000
CLSTN1	Calsyntenin-1		Proteintech	#12788-1-AP	rabbit	1:1000
COL5A1	Collagen alpha-1(V) chain		santa cruz	#sc-166155	mouse	1:1000
COL8A1	Collagen alpha-1(VIII) chain		Proteintech	#17251-1-AP	rabbit	1:1000
COL18A1	Collagen alpha-1(XVIII) chain		Proteintech	#18301-1-AP	rabbit	1:1000
DAG1	α-Dystroglycan		santa cruz	#sc-53987	mouse	1:1000
DAG1	β-Dystroglycan		santa cruz	#sc-33702	mouse	1:1000
ENG	Endoglin		Proteintech	#10862-1-AP	rabbit	1:1000
EpCAM	EpCAM		Abcam	#ab71916	rabbit	1:1000
FAK	Focal adhesion kinase 1	Y397	Abcam	#ab24781	mouse	1:100
FERMT2	Kindlin-2		Proteintech	#11453-1-AP	rabbit	1:2000
FN1	Fibronectin		santa cruz	#sc-8422	mouse	1:500
GAPDH	Glyceraldehyde-3-phosphate dehydrogenase		santa cruz	#sc-32233	mouse	1:2000
GJA1	Connexin-43		Proteintech	#26980-1-AP	rabbit	1:1000
GJC1	Connexin-45		santa cruz	#sc-374354	mouse	1:1000
GOLGA2	GM130		CST	#12480	rabbit	1:1000
GPC1	Glypican-1		Proteintech	#16700-1-AP	rabbit	1:1000
GPC4	Glypican-4		Proteintech	#13048-1-AP	rabbit	1:500
HDAC1	Histone deacetylase 1		CST	#2062	rabbit	1:1000
HSPG2	Perlecan		Proteintech	#19675-1-AP	rabbit	1:1000
HSP90AA1/B1	HSP90		santa cruz	#sc-13119	mouse	1:1000
IGF1R	Insulin-like growth factor 1 receptor		Proteintech	#20254-1-AP	rabbit	1:1000
IGSF8	Immunoglobulin superfamily member 8		Proteintech	#14387-1-AP	rabbit	1:1000
ITGA3	Integrin alpha-3		Proteintech	#66070-1-Ig	mouse	1:1000
ITGAV	Integrin alpha-V		santa cruz	#sc-376156	mouse	1:1000
ITGB3	Integrin beta-3		santa cruz	#sc-365679	mouse	1:1000
LAMA5	Laminin subunit alpha-5		Thermo Fisher	#PA5-49930	rabbit	1:1000
LAMP2	Lysosome-associated membrane glycoprotein 2		Thermo Fisher	#PA1-655	rabbit	1:100
MSLN	Mesothelin		CST	#65367	rabbit	1:1000
NECTIN2	Nectin-2		santa cruz	#sc-271236	mouse	1:1000
NPTN	Neuroplastin		Proteintech	#28022-1-AP	rabbit	1:1000
RGAP1	Rac GTPase-activating protein 1		santa cruz	#sc-271110	mouse	1:1000
SDCBP	Syntenin-1		abcam	#ab133267	rabbit	1:2000
SDC4	Syndecan-4		abnova	#PAB9045	rabbit	1:500
SRC	Proto-oncogene tyrosine-protein kinase Src	Y416	CST	#6943T	rabbit	1:100
TLN1	Talin-1		santa cruz	#sc-365875	mouse	1:1000
TSG101	Tumor susceptibility gene 101 protein		santa cruz	#sc-7964	mouse	1:1000
VCAM1 (h)	Vascular cell adhesion protein 1		santa cruz	#sc-13160	mouse	1:250
VCAM1 (m)	Vascular cell adhesion protein 1		Proteintech	#83719-1-RR	rabbit	1:1000

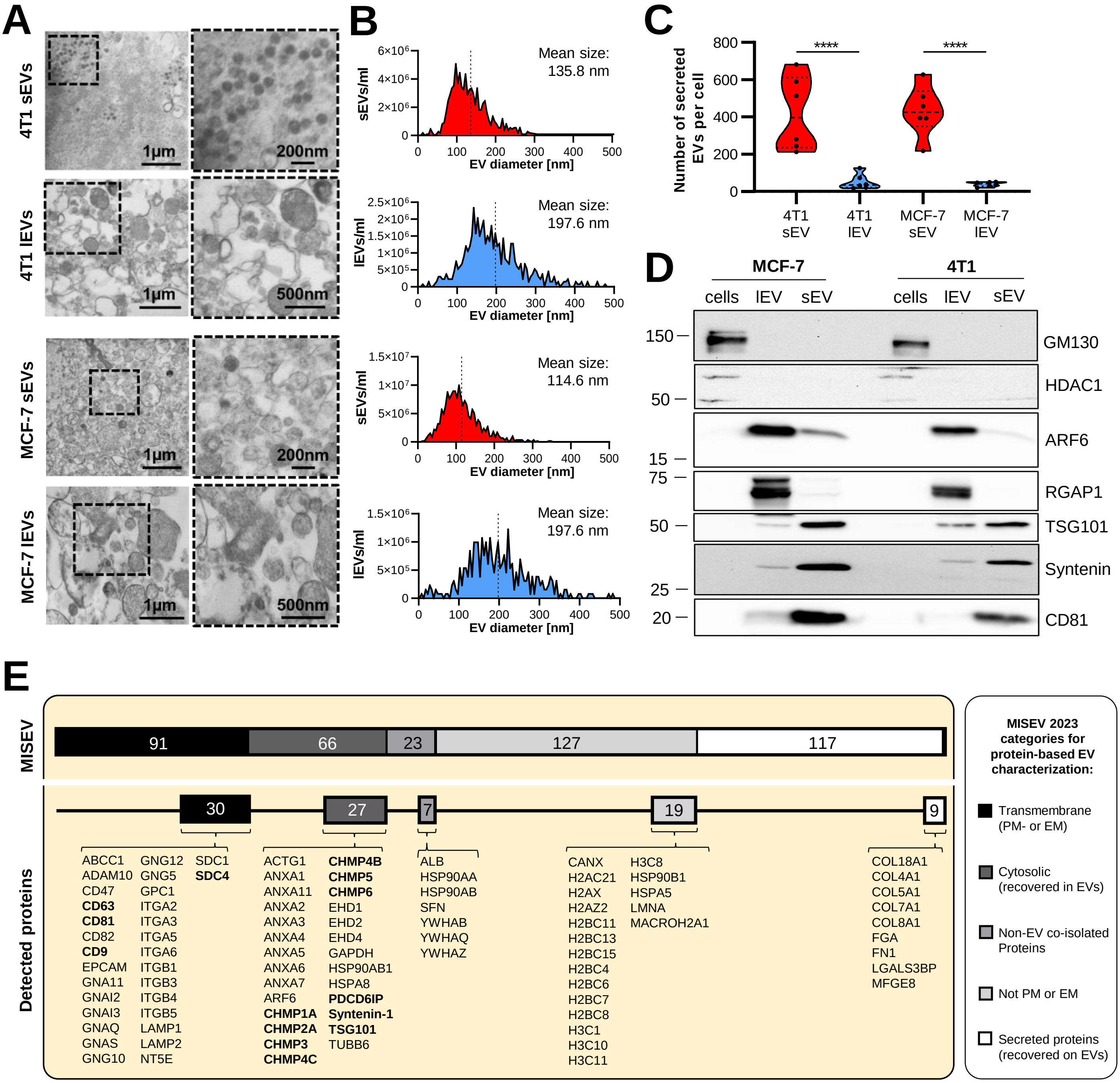

Supplementary Figure 1: Characterizarion of breast cancer-derived EVs. A, TEM images of lEVs and sEVs isolated from 4T1 and MCF-7 cells. **B+C,** NTA of EVs isolated from 4T1 and MCF-7 cells. Shown are representative size profiles (B) and the numbers of secreted lEVs and sEVs per cell at the day of EV isolation (C). **D,** Immunoblot analysis: Expression of selected EV positive and negative markers on EVs from 4T1 and MCF-7 cells. For all conditions, the same protein amount was loaded on the gel (10 µg). **E,** Overlap between proteins of the different MISEV 2023 categories for protein-based EV characterization and the proteins on 4T1 sEVs detected by mass spectrometry. PM: Plasma membrane, EM: Endosomal membrane.

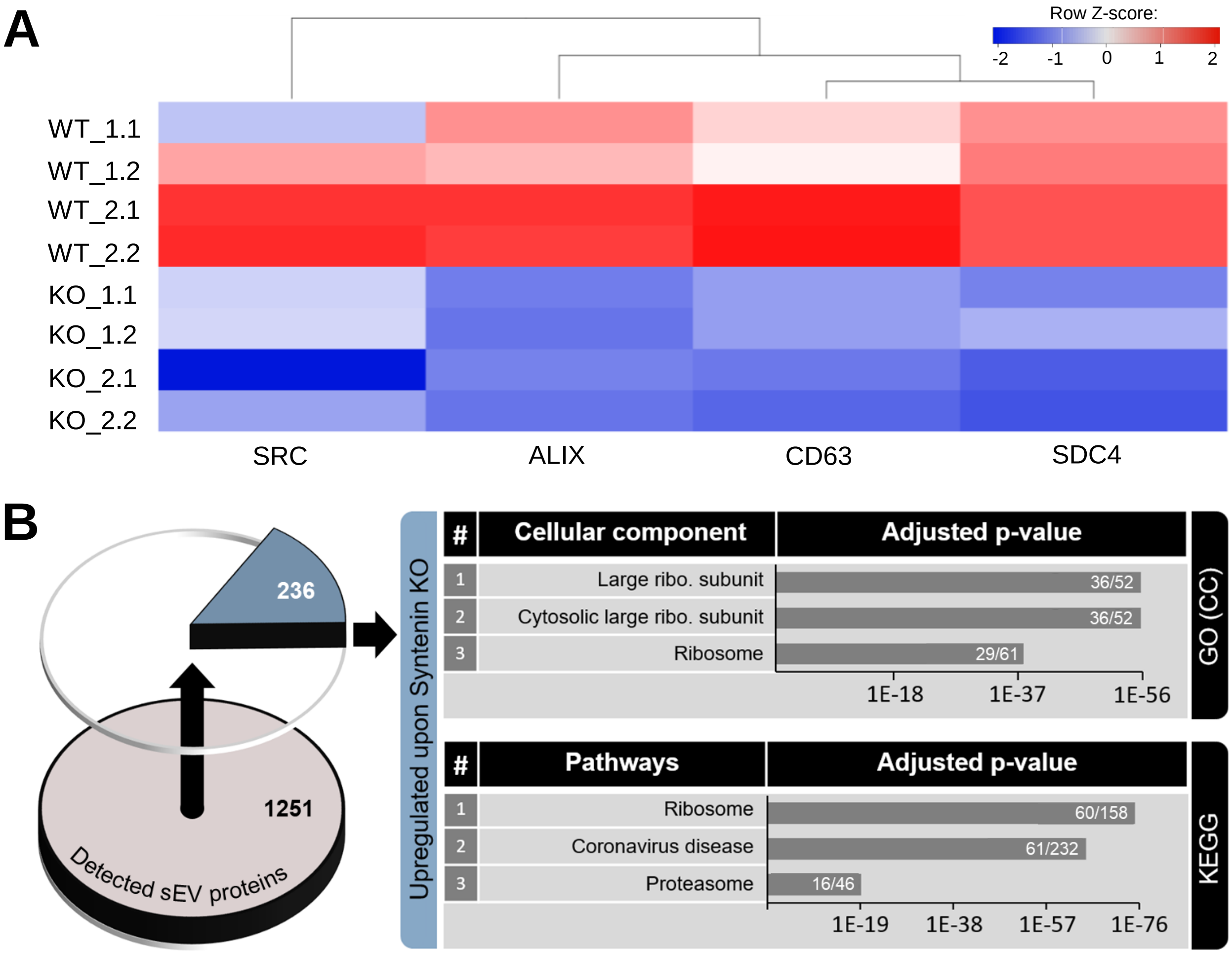

Supplementary Figure 2: Syntenin loss-of-funtion leads to a decreased expression of known binding partners and increased levels of ribosomal proteins on sEVs. A, Mass spectrometry: Selective heatmap showing the expression of known Syntenin binding partners on sEVs from 4T1 WT and Syntenin knockout (KO, KO_1/2: 2 clones) cells. **B,** Pathway enrichment analysis for all 236 sEV proteins significantly up-regulated ($p<0.05$) upon Syntenin KO. Shown are the top three associations based on the Gene Ontology (GO) (Cellular Component (CC)) and Kyoto Encyclopedia of Genes and Genomes (KEGG) database. The numbers given in the individual bars indicate the number of proteins identified in the mass spectrometry analysis in relation to the total number of proteins in the respective pathway.

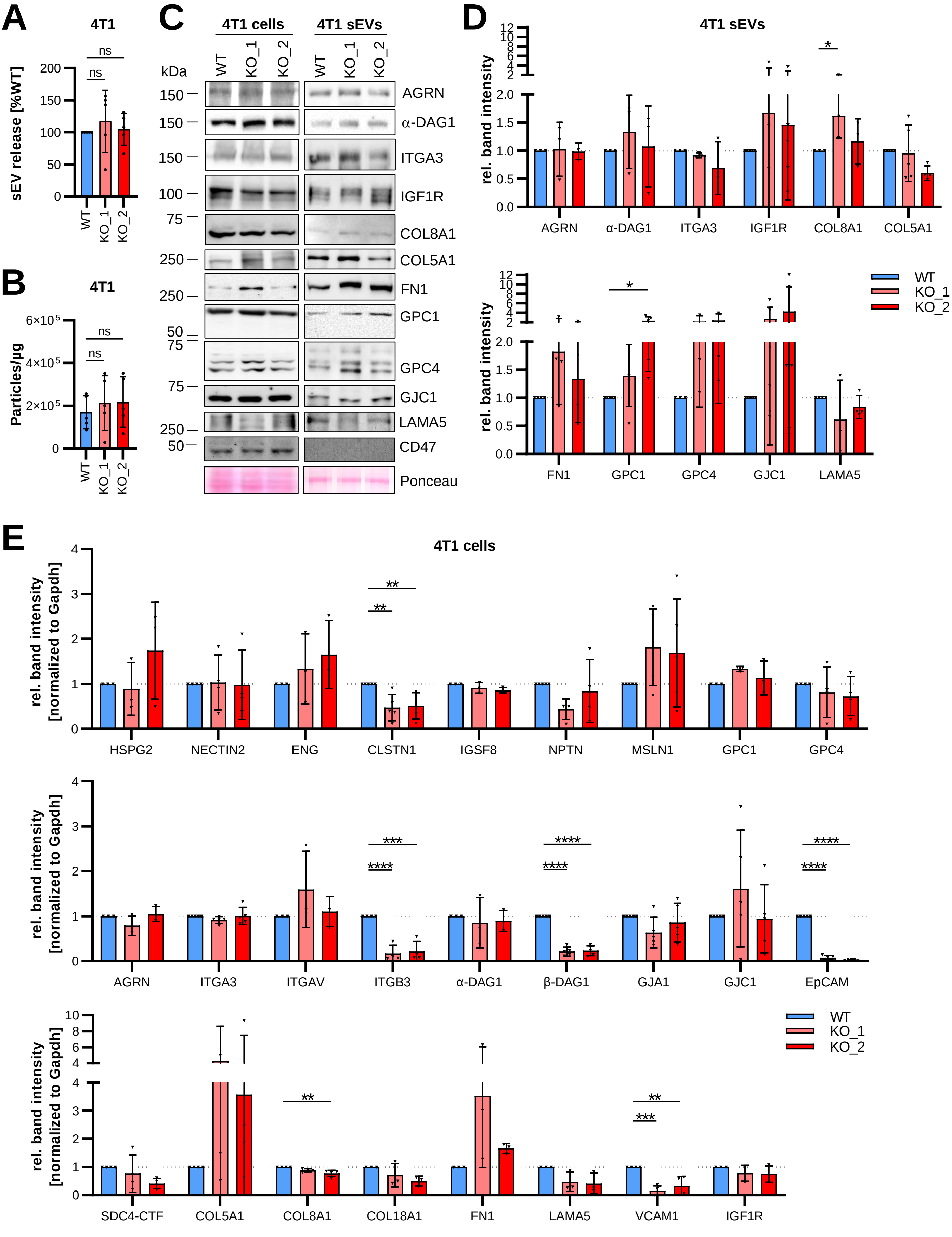

Supplementary Figure 3: Expression of adhesion proteins in 4T1 cell lysates and sEVs upon Syntenin knock-out. A, The number of sEVs released from 4T1 WT and Syntenin knockout (KO, KO_1/2: 2 clones) cells was quantified by NTA (mean±SD). **B**, Ratio of particles per µg EV protein (mean±SD). **C+D,** The expression of the indicated adhesion proteins was analyzed in 4T1 cell lysates and sEVs by immunoblot (C) and signals in sEVs quantified by densitometry (mean±SD) (D). The same amount of protein was loaded for siCTL and KD samples. **E,** The expression of the indicated adhesion proteins had been analysed by immunoblots and signals in 4T1 cell lysates were quantified by densitometry (mean±SD).

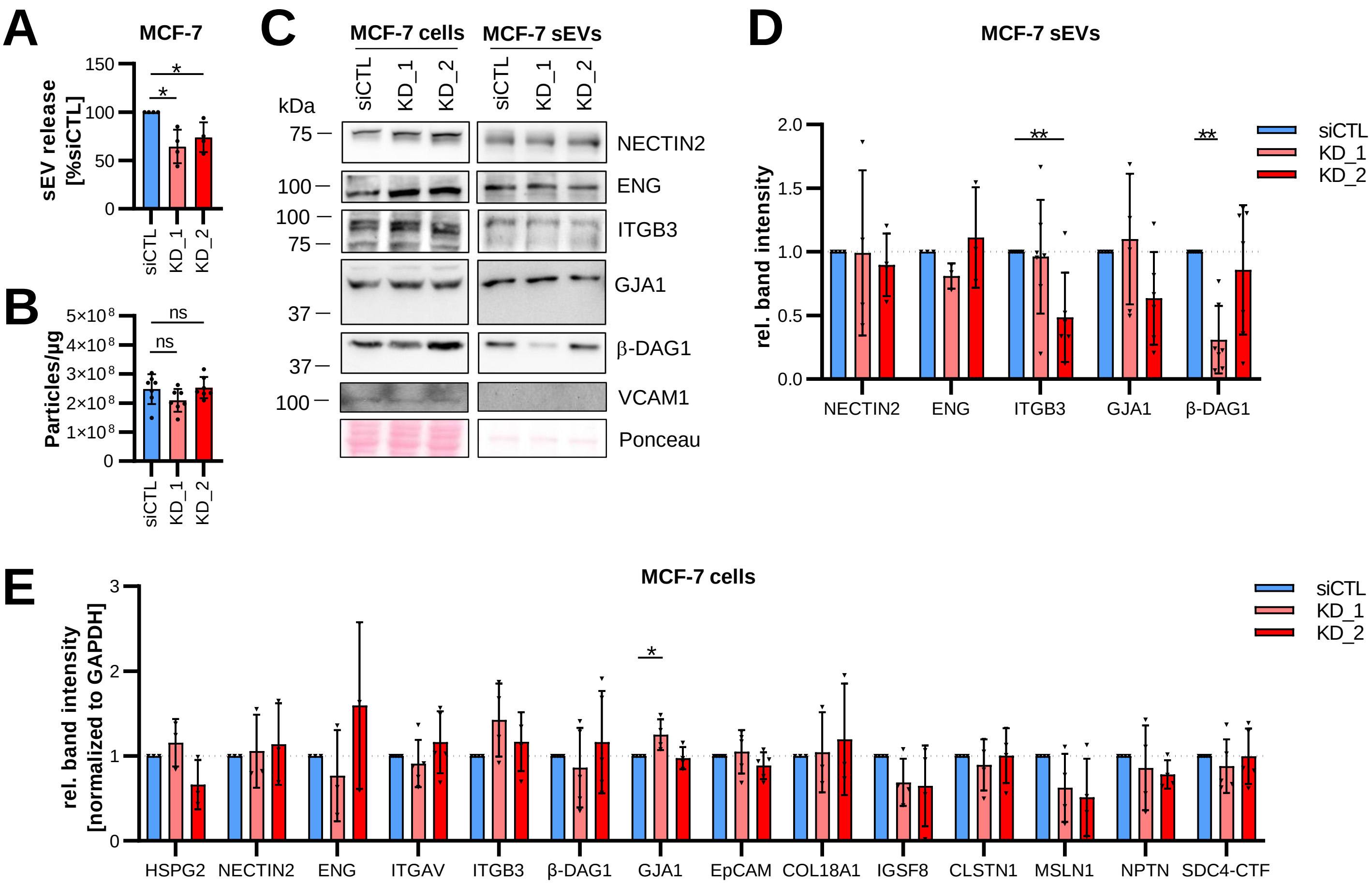

Supplementary Figure 4: Expression of adhesion proteins in MCF-7 cell lysates and sEVs upon Syntenin loss-of-function. A, The number of sEVs released from MCF-7 siCTL and Syntenin knockdown (KD, KD_1/2: 2 siRNA sequences) cells was quantified by NTA (mean±SD). **B**, Ratio of particles per µg EV protein (mean±SD). **C+D,** The expression of the indicated adhesion proteins was analyzed in MCF-7 cell lysates and sEVs by immunoblot (C) and signals in sEVs quantified by densitometry (mean±SD) (D). The same amount of protein was loaded for siCTL and KD samples. **E,** The expression of the indicated adhesion proteins had been analysed by immunoblots and signals in MCF-7 cell lysates were quantified by densitometry (mean±SD).

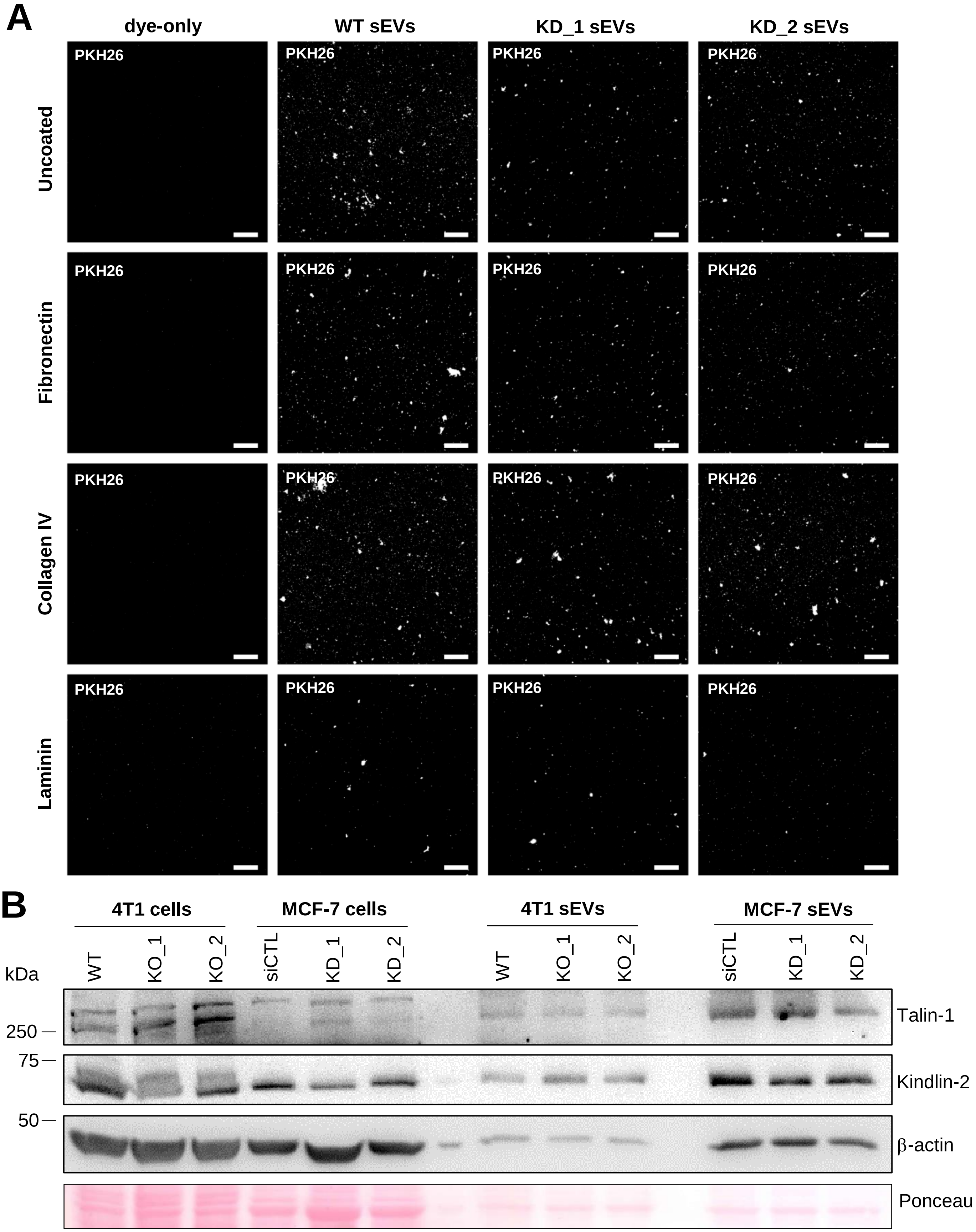

Supplementary Figure 5: Syntenin regulates the Fibronectin-binding capacity of breast cancer-derived sEVs but has no effect on the expression of Integrin activators. A, Confocal microscopy: sEVs isolated from MCF-7 cells treated with a control siRNA (siCTL) or siRNA for Syntenin (KD_1/2: 2 sequences) were labeled with PKH26 and seeded on uncoated and Fibronectin-, Collagen IV- or Laminin-coated glass coverslips. Dye-only samples served as controls. Scale bar: 20 µm. **B,** Immunoblots: Expression of the Integrin activators Talin-1 and Kindlin-2 in cell lysates and sEVs from 4T1 and MCF-7 cells with Syntenin knockout (KO_1/2: 2 clones) or KD. Same protein amounts of lysates (50 µg) and sEVs (10 µg) were loaded onto the gels. β-actin served as housekeeping protein.

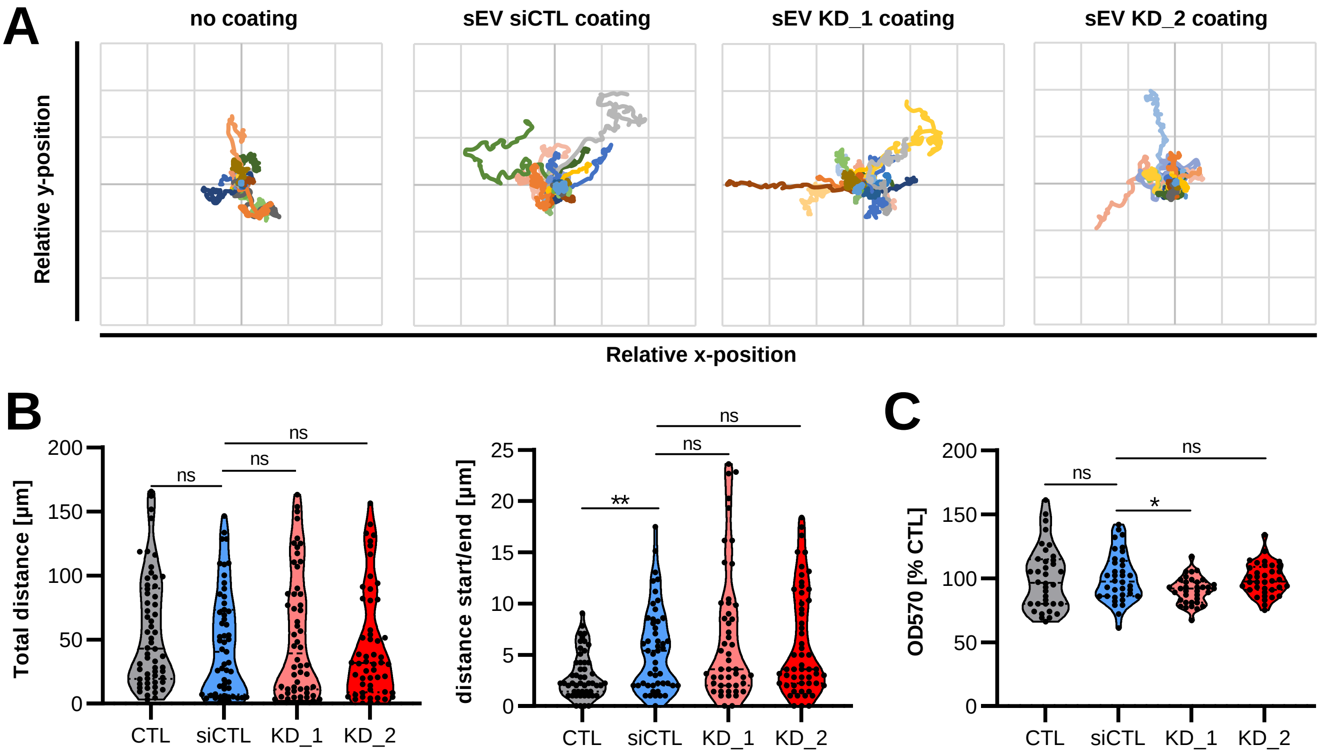

Supplementary Figure 6: MCF-7 sEVs exhibit weak pro-migratory effects without a significant impact of Syntenin loss-of-function. A+B, MCF-7 cells were seeded on top of sEVs isolated from cells treated either with a control siRNA (siCTL) or Syntenin knockdown (KD_1/2: 2 sequences) and were tracked for 8 h by live cell imaging. Based on the manually tracked migration of individual cells (*A*), the total migration distance as well as the distance between start and end point were calculated (*B*). Data is shown as spider plots for relative cell movements and violin plots showing median, lower and upper quartiles (n=60). **C,** Cell adhesion assay: MCF-7 cells were seeded for 10 min on plates pre-coated with WT or Syntenin KD sEVs. Data is represented as violin plots showing median, lower and upper quartiles (n=30).

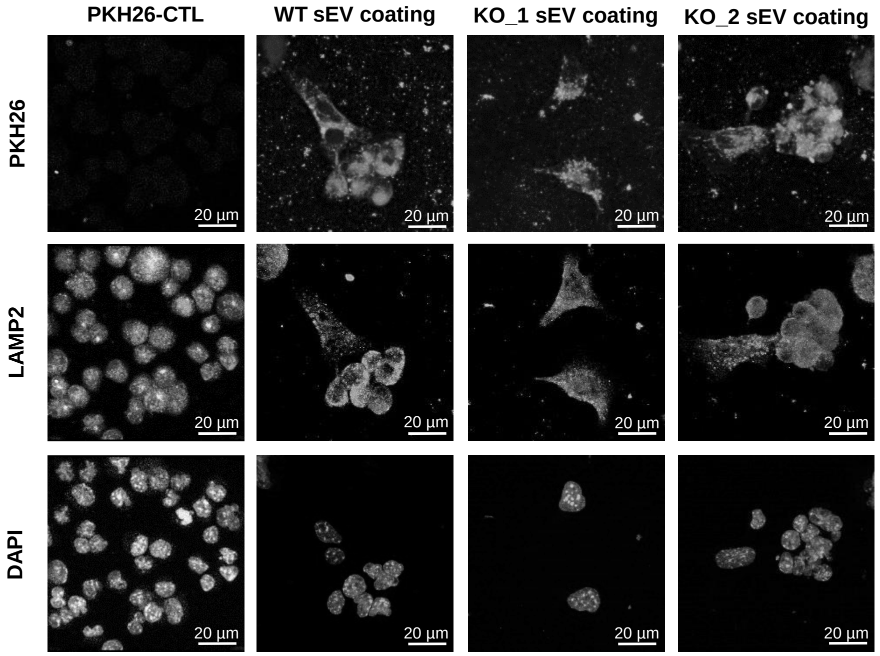

Supplementary Figure 7: Uptake of tumor-EVs into 4T1 cells. 4T1 cells were seeded on top of PKH26-labeled WT or Syntenin KO (KO_1/2: 2 clones) sEVs and stained for the late endosomal marker LAMP2. Uptake of sEVs into the tumor cells was visualized by confocal microscopy. Overlay images and representative Z stacks are found in Fig. 5A.

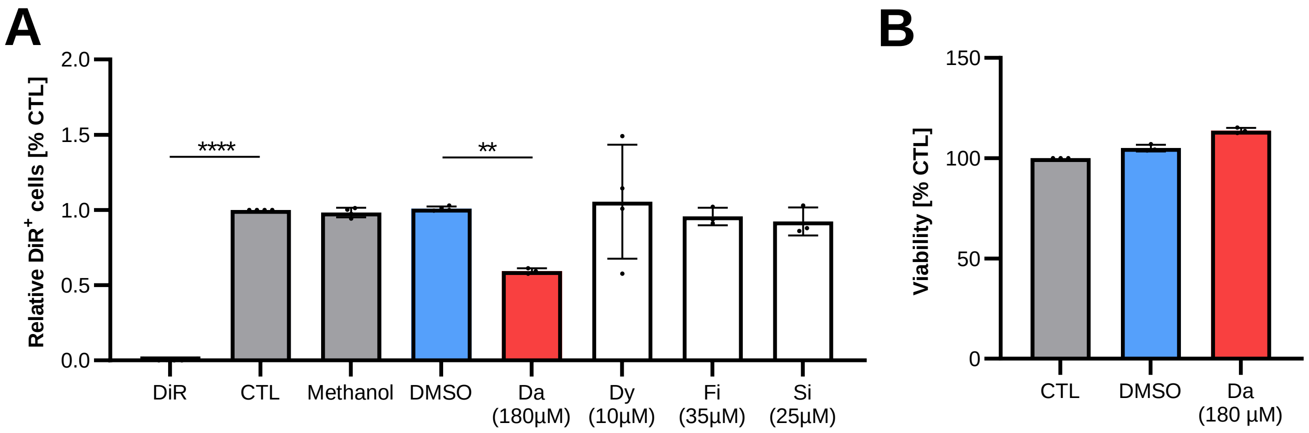

Supplementary Figure 8: Dansycadaverine inhibits EV uptake in 4T1 cells. A, Flow cytometry: 4T1 cells were pre-treated with Dansylcadaverine (Da, 180 µM), Dynasore (Dy, 10 µM), Filipin III (Fi, 35 µM), Simvastatin (Si, 25 µM) or solvent controls and subsequently exposed to DiR-labeled 4T1 sEVs or dye-only controls (DiR). The percentage of DiR-positive cells was normalized to the values of untreated cells (CTL) (mean±SD). **B,** MTT assay: Viability of 4T1 cells after 24 h of exposure to Da (180 µM) or DMSO (1:1035) (mean±SD).
